# Predicted structural proteome of *Sphagnum divinum* and proteome-scale annotation

**DOI:** 10.1093/bioinformatics/btad511

**Published:** 2023-08-17

**Authors:** Russell B Davidson, Mark Coletti, Mu Gao, Bryan Piatkowski, Avinash Sreedasyam, Farhan Quadir, David J Weston, Jeremy Schmutz, Jianlin Cheng, Jeffrey Skolnick, Jerry M Parks, Ada Sedova

**Affiliations:** Biosciences Division, Oak Ridge National Laboratory, Oak Ridge, TN 37830, United States; Computer Science and Mathematics Division, Oak Ridge National Laboratory, Oak Ridge, TN 37830, United States; Center for the Study of Systems Biology, School of Biological Sciences, Georgia Institute of Technology, Atlanta, GA 30332, United States; Biosciences Division, Oak Ridge National Laboratory, Oak Ridge, TN 37830, United States; Genome Sequencing Center, HudsonAlpha Institute for Biotechnology, Huntsville, AL 35806, United States; Electrical Engineering and Computer Science, University of Missouri, Columbia, MS 65211, United States; Biosciences Division, Oak Ridge National Laboratory, Oak Ridge, TN 37830, United States; Genome Sequencing Center, HudsonAlpha Institute for Biotechnology, Huntsville, AL 35806, United States; Department of Energy Joint Genome Institute, Lawrence Berkeley National Laboratory, Berkeley, CA 94720, United States; Electrical Engineering and Computer Science, University of Missouri, Columbia, MS 65211, United States; Center for the Study of Systems Biology, School of Biological Sciences, Georgia Institute of Technology, Atlanta, GA 30332, United States; Biosciences Division, Oak Ridge National Laboratory, Oak Ridge, TN 37830, United States; Biosciences Division, Oak Ridge National Laboratory, Oak Ridge, TN 37830, United States

## Abstract

**Motivation:**

*Sphagnum*-dominated peatlands store a substantial amount of terrestrial carbon. The genus is undersampled and under-studied. No experimental crystal structure from any *Sphagnum* species exists in the Protein Data Bank and fewer than 200 *Sphagnum*-related genes have structural models available in the AlphaFold Protein Structure Database. Tools and resources are needed to help bridge these gaps, and to enable the analysis of other structural proteomes now made possible by accurate structure prediction.

**Results:**

We present the predicted structural proteome (25 134 primary transcripts) of *Sphagnum divinum* computed using AlphaFold, structural alignment results of all high-confidence models against an annotated nonredundant crystallographic database of over 90,000 structures, a structure-based classification of putative Enzyme Commission (EC) numbers across this proteome, and the computational method to perform this proteome-scale structure-based annotation.

**Availability and implementation:**

All data and code are available in public repositories, detailed at https://github.com/BSDExabio/SAFA. The structural models of the *S. divinum* proteome have been deposited in the ModelArchive repository at https://modelarchive.org/doi/10.5452/ma-ornl-sphdiv.

## 1 Introduction

*Sphagnum*-dominated peatlands, threatened by warming climates, convert and store about 25%–30% of global terrestrial carbon, making them critically important atmospheric carbon sinks (Healey *et al.* 2023). Genetic and phylogenomic studies of the newly recognized *Sphagnum magellanicum* complex, which contains *Sphagnum* species that are widespread in global peatland ecosystems, have recently resolved genomic divergence, suggesting these plants may be actively speciating (Shaw *et al.* 2022). The newly sequenced genome of the *Sphagnum divinum* species from this complex is of reference quality and represents an essential tool for ecological and genomic research on peat mosses and peatland conservation (Weston *et al.* 2018, Shaw *et al.* 2022). However, *Sphagnum* species remain an undersampled and under-studied lineage of land plants, from both the evolutionary (Shaw *et al.* 2022, Healey *et al.* 2023) and structural biology standpoints. Protein structure can provide crucial information about protein function, stability, molecular interactions, effects of mutations, biochemical mechanisms, and many other properties; however, within the Protein Data Bank (PDB) (Berman *et al.* 2000), fewer than 40 total structures from any bryophytes are found, with none from any *Sphagnum* species.

Consistent breakthroughs in protein structure prediction over the past decade culminated in extremely high accuracy predictions obtained from the second version of AlphaFold (Jumper *et al.* 2021) and produced the AlphaFold Protein Structure Database (AlphaFold DB) (Varadi *et al.* 2022), which covers predicted models of the proteins found in the UniProt UniRef90 dataset (over 200 million proteins) (Consortium 2023). The AlphaFold DB currently includes 48 proteome-scale datasets of predicted structures. Prior to this development, proteome-scale structural analysis was unprecedented (Tunyasuvunakool *et al.* 2021). Since the *S. divinum* genome is a recent result initially released in the JGI Phytozome catalog (Goodstein *et al.* 2012), AlphaFold DB does not include models associated with this species. The use of predicted structure to help understand protein function is especially important in *Sphagnum* species, as gene characterization through transformation does not currently exist.

In recent studies of the response of *S. divinum* to environmental stressors, complex interactions were found between sex genes, autosomal genes, and environmental conditions (Healey *et al.* 2023). Protein structural information is expected to provide missing details in regard to understanding pathways and mechanisms related to relevant encoded proteins. Structural alignment may provide a more sensitive way to detect remote homologs, while 3D molecular details can help uncover biochemical information such as binding pockets and catalytic residues. The challenge is to determine the confidence in the assignment of function when structures are similar, especially when alignments are performed across kingdoms or phyla. Here, we refer to the assignment of specific biological functions, represented by classification ontologies, families, or indices such Enzyme Commission (EC) numbers (Bairoch 2000) as functional annotation, to differentiate it from annotation involving identification of protein coding sequences. In the latter case, the automation of annotating genes with functional metadata may be incorrect, and errors can propagate when incorrect assignments are transferred from proteome to proteome (Rembeza and Engqvist 2021). Additional information that can support or refute computational annotations is needed.

Here, we present the predicted structures of 25,134 proteins from the *S. divinum* proteome (representing the majority of the primary transcripts, except for long protein sequences with more than 2,500 residues), computed using AlphaFold 2 with a dynamical recycling approach and an optimized clash-reducing refinement step (Gao *et al.* 2022). This structural proteome adds to the existing collection of new organism-scale structural datasets such as the model-organism proteomes found in AlphaFold DB. We use this structural proteome to develop a computational approach for proteome-scale structure-based functional annotation of enzymes: 3D structural alignment of all high-quality predicted models to the nonredundant set of experimentally determined protein structures, followed by a consensus classification and an analysis of conserved active sites and binding sites. In developing this method, we have also produced an annotated version of the PDB70 structural database that contains aligned residue-level information on active sites and binding sites used in our method. We have incorporated parallel programming techniques in the implementation of this method to produce an efficient and scalable pipeline. We apply this method to the high-quality structural models of the *S. divinum* proteome, returning new annotation information for a set of stress–response-related proteins, for proteins with no sequence-based functional annotations, and to complement the remainder of the proteins for which some sequence-homology-based computational annotation was previously derived. We highlight the novel findings that this type of approach can produce with several important examples from the set of *S. divinum* stress-response proteins.

## 2 Materials and methods

### 2.1 Overview of the structure-based annotation pipeline

**Figure 1.**
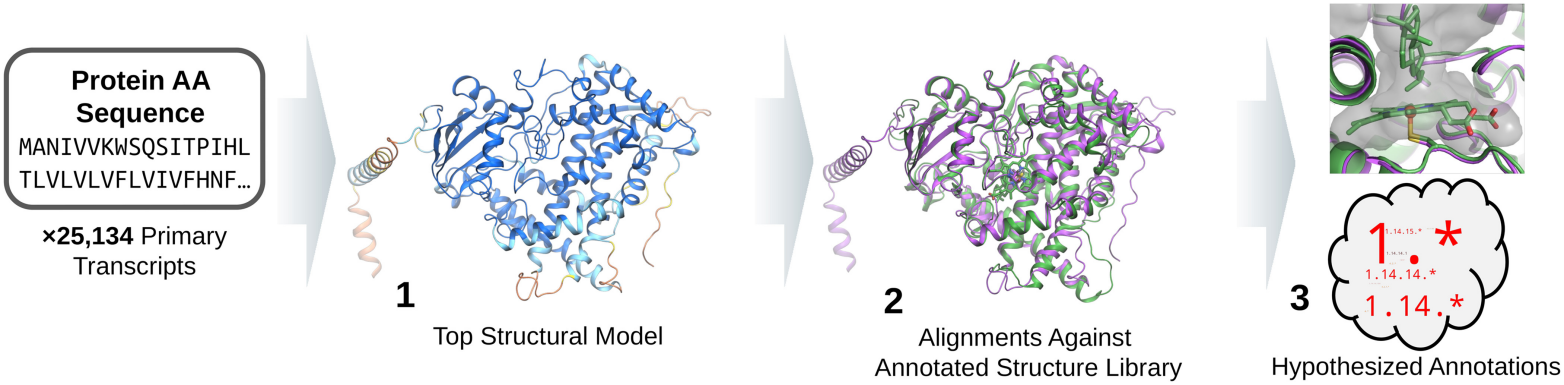
Schema of structure prediction, alignment, and hypothesis development for functional annotation. (1) For each of the 25,134 primary transcripts in the *S. divinum* proteome, five structural models are inferred using AlphaFold2 with structures ranked using the pTM score. The top ranked structural model is used in structural alignments (2) against a library of experimental structures (e.g. PDB70 structural library, reported here) to identify strong structural analogs. Metadata and structural feature annotations from structural alignment hits are gathered and parsed (3) to provide a hypothesis as to the functional annotation for the modeled protein, such as Enzyme Commission number (EC) and residue-level structural insights, using a relative entropy-based classification which considers the ensemble of well-aligned structural matches.

Figure 1 depicts the schema of the structural alignment for functional annotation (SAFA) workflow. First, predicted structural models are generated. Both the predicted template modeling score (pTM) and average predicted local distance difference test (pLDDT) scores are used to judge the model quality: if either the pTM > 0.7 or the pLDDT > 70, the model is passed on to step 2 in Fig. 1, the structural alignment against a library of annotated, experimental structures to identify structural analogs. Finally, annotations from the structural alignment hits are gathered, parsed, and suggested as hypotheses for the modeled protein’s function, as shown in Step 3 of Fig. 1. The methods utilized for this workflow are described below and are more thoroughly covered in the Supplementary Information (SI), Supplementary Section S2.

### 2.2 Proteome-scale structure prediction with AlphaFold

Structure prediction for the set of primary transcripts of the *S. divinum* genome (NCBI Taxonomy ID 128215, available on JGI Phytozome) was performed using AlphaFold Monomer v2 (Jumper *et al.* 2021) on the Andes and Summit supercomputers at the Oak Ridge Leadership Computing Facility, as described previously (Gao *et al.* 2022). Of the 25,227 primary transcripts of the *S. divinum* genome, 25,134 structures were predicted. Sequences with more than 2,500 residues were not modeled due to hardware limitations.

### 2.3 Annotated PDB70 structural library

Structures corresponding to PDB accession and chain IDs listed in the PDB70 sequence database (dated 13 March 2022) (Steinegger *et al.* 2019) were used for creating the annotated structural library, totaling 91,032 single-chain protein structures. In addition to gathering the structures, we also collect the annotation metadata from UniProt flat files associated with each structure in the PDB70 structural library. For this study, residue-specific annotations categorized as active site and binding site features were retrieved using our annotation pipeline. EC numbers were also gathered, when available.

### 2.4 Structural alignments

US-align2 was used to perform structural alignments between *S. divinum* protein models and the PDB70 structural library using the semi-nonsequential (sNS) alignment algorithm (Zhang and Pyle 2022) with a parallel workflow.

### 2.5 Hypothesis development for annotation of enzymes

Structures that strongly align to an inferred model are gathered and considered as a set. The metadata data associated with these structures is parsed to identify agreements in qualitative data (e.g. EC numbers) as well as structural features such as ligand binding or active site residues using consensus methods. The enzymatic annotation hypothesis is generated in two steps, using an analysis of the EC numbers found in the hits’ metadata: (i) the classification of the protein as an enzyme and (ii) a consensus classification resulting in the assignment of either an EC number, whether a full four digit label (e.g. 1.1.1.1) or a prefix (e.g. 1.${}^{*}$, 1.1.${}^{*}$, 1.1.1.${}^{*}$), or the label “no consensus” to the protein. The active and binding site residue-level feature metadata are then used to support the EC number hypothesis by highlighting the important residues in the predicted model that may play key roles in the proposed enzymatic function.

#### 2.5.1 Assigning EC numbers using relative entropy

To determine whether a structural model can be classified as an enzyme, the EC numbers observed in the model’s set of alignment hits are compared to the background distribution of EC numbers observed in the full PDB70 structural library. Specifically, the Kullback–Leibler divergence ($D_{KL}$), also called the relative entropy, is calculated as follows: which represents the information gain from the probability distribution, *P*, of the EC numbers from the model’s set of alignment hits relative to the background distribution, *Q*, of EC numbers in the PDB70 structural library, where the set of EC numbers observed in the library is the discrete sample space, *X*. Further details about this metric and the statistical significance test are found in Supplementary Section S2.4 in the SI.


(1) $$D_{KL}(P∥Q)=\sum_{x\in X}P(x){\;\text{log}\;}_{2}\left(\frac{P(x)}{Q(x)}\right)$$

In the second stage of the annotation process, the relative entropy components (the terms within the summation in Equation 1) associated with each observed EC number, *x*, are considered, to determine which, if any, EC number should be assigned to the model as the annotation label. A strict majority consensus rule is applied: if one EC number’s relative entropy component represents 50% or more of the total relative entropy value, then that EC number is the primary enzyme annotation.

#### 2.5.2 Residue-level insights

The UniProt flat files may contain information at the residue-level, highlighting a large variety of structural features. For this work, focus is given to “BINDING” and “ACT_SITE” feature labels. To highlight importance of specific residue positions in the structural model as well as guide visualizations, the number of times a model’s residues are aligned to residues in a PDB70 structure that are associated with either type of feature element is counted; this counting metric is discussed as the “feature count.” The “conservation count” quantifies the number of instances in which both model and alignment target have the *identical* residue type at the structurally aligned position. In this way, we can begin to localize active or binding sites based on the ensemble of alignment hits’ annotation information.

### 2.6 Sequence alignments

We benchmarked the structural alignment method against established sequence-based methods designed for finding remote structural homologs. Further details and discussion are found in Supplementary Sections S2.6 and S4.

### 2.7 Transcriptomic response to heat shock experiments

New gene expression analysis experiments were performed to validate the transcriptomic responses of Sphmag01G194900, Sphmag02G160700, and Sphmag13G047200 to heat shock, which were first reported in Healey *et al.* (2023). Further details are found in Supplementary Section S2.7 of the SI.

### 2.8 Data Availability

All data and code are available in public repositories, detailed at https://github.com/BSDExabio/SAFA. The structural models of the S. divinum proteome have been deposited in the ModelArchive repository at https://modelarchive.org/doi/10.5452/ma-ornl-sphdiv.

## 3 Results

As previously mentioned, no experimental structures for *Sphagnum* species exist in the PDB. The AlphaFold DB holds 5007 models across all *Sphagnum* species, whether the species is named or currently unclassified. These models represent structures of only 143 unique genes, as counted by “gene name” annotations in the associated UniProtKB flat files (structure list parsed on 16 March 2023). To put this number into context, *S. divinum* encodes 25,227 primary transcripts; the 143 unique, nonhomologous proteins represented in AlphaFold DB only account for 0.5% of the encoded proteome. To further analyze the *S. divinum* proteome, we have modeled the structures of a majority of the primary transcripts and used the structural alignment pipeline, illustrated in Fig. 1, to identify analogous, annotated protein structures. Structural alignment results are then used to develop hypotheses for protein function as well as support results obtained from experiments and other computational annotation methods.

### 3.1 Proteome-scale structure predictions

Supplementary Figure S1 shows the distribution of average pLDDT scores of *S. divinum* models alongside distributions from AlphaFold DB’s reference proteomes of *Oryza sativa* (rice), *Zea mays* (corn), *Arabidopsis thaliana* (thale cress), *Glycine max* (soybean), *Mus musculus* (mouse), and *Homo sapiens* (human). In this context, the *S. divinum* model quality distribution mirrors those of the reference proteomes, with roughly 57% of predicted structures being well-modeled by AlphaFold, using the average pLDDT score cutoff of 70 as suggested on the AlphaFold DB website. For *S. divinum* and *O. sativa*, there is increased density in the lower-confidence, average pLDDT score region than for the other proteomes. These low confidence structural models observed in *S. divinum* may represent incorrectly-annotated sections of the genome that may not encode proteins, intrinsically disordered proteins (IDPs) (Ruff and Pappu 2021, Akdel *et al.* 2022), or potential weaknesses in the AlphaFold inference model. As alluded to above, initial gene annotation methods may identify primary transcripts in the genome that are not expressed *in vivo*. Alternatively, IDPs serve important roles in plants, including in response to drought stress (Balcerowicz 2020); these important proteins will not be modeled well by AlphaFold. Finally, paucity of proteins originating from plants, and more specifically bryophytes, in the AlphaFold training data may result in a bias toward low confidence scores for predicted structures of proteins from moss genomes.

**Figure 2.**
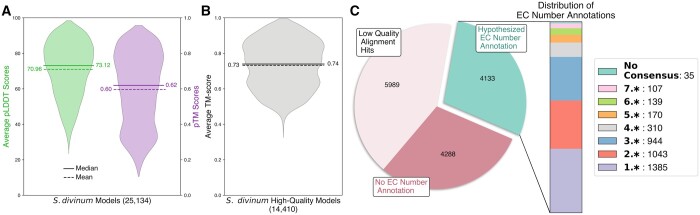
Proteome-scale distributions for *S. divinum*. (A) AlphaFold pTM and pLDDT model quality scores for all modeled *S. divinum* primary transcripts. If either pTM >0.7 or average pLDDT >70, then the model is considered high-quality. (B) The distribution of high-quality structural models’ top-ranking average TM-score value from alignments to the PDB70 structural library. (C) Breakdown of the structural alignment results into categories for models without any strong alignment hits, with a non-EC number annotation hypothesis, or with an enzymatic annotation hypothesis. For the last category, the stacked bar depicts the distribution of first digit EC numbers hypothesized for the respective category as well as the small number of cases where no consensus EC number was obtained.

Figure 2A compares the model quality distributions for the average pLDDT and pTM metrics for the *S. divinum* proteome structures. Median values are 73.12 and 0.62, respectively. Using average pLDDT as the lone model quality score can be misleading: the average pLDDT score can be a poor descriptive statistic when large unstructured loops are given equal weight as well-modeled structural domains. On the other hand, the pTM score may quantify a higher confidence result for models with a globular protein structure that are plagued by poorly modeled termini or loop regions. When available, both average pLDDT and pTM scores should be considered. For the *S. divinum* proteins, there are 14,410 primary transcript models that pass our model quality criteria of pTM > 0.7 or average pLDDT > 70.

### 3.2 Structural alignment for functional annotation

The set of confident *S. divinum* structural models (those that satisfy the model quality criteria) are forwarded to the structural alignment step of the workflow, where each protein model is aligned to all structures in the PDB70 structural library (91,032 structures), using the sNS method of US-align2. In total, this equates to approximately 1.3 billion structure alignment calculations, the results of which are provided as a publicly available dataset described further in Supplementary Section S1 of the SI. Figure 2B shows the distribution of each model’s largest average TM-score, which is the metric used as the criterion for alignment ranking. The 5,969 models with their highest-ranking alignment’s average TM-score < 0.7 are not considered for development of an annotation hypothesis (step 3 in Fig. 1; see panel C of Fig. 2). However, these structures may still be of interest as novel domain assemblies, potentially representing new structural motifs not yet found or poorly represented in the existing experimental data. For example, there are 266 models that have either average pLDDT or pTM scores > 90 or 0.9, respectively, with no structural alignment hits based on the 0.7 average TM-score cutoff. Investigation of these high-confidence structural models and many others may provide insights including the potential discovery of new domain arrangements or new folds. The remaining 8,421 models of *S. divinum* proteins have at least one structural alignment hit in the PDB70 structural library, from which we hypothesize annotations and residue-level insights can be transferred to the model. Comparison of the structural alignment method against a state-of-the-art sequence-based alignment method is presented Supplementary Section S4 in the SI.

#### 3.2.1 Proteome-scale structure-based enzyme annotation

To develop annotation hypotheses for the 8,421 models that align well to one or more structures in the PDB70 structural library, the metadata pipeline is used to retrieve relevant qualitative information and all residue-level features found in the metadata file from the UniProtKB entries corresponding to the matching crystal structure(s). Here, we focus on identifying enzymes and labeling those protein models with an appropriate EC number.

To initially develop an enzyme annotation for a protein, a statistical analysis is performed on the set of EC numbers gathered from a model’s structural alignment hits to propose whether the modeled protein is an enzyme or not. If the modeled protein is hypothesized to be enzymatic, the second stage of the analysis uses a consensus classification to assign a primary EC number to that model. As shown in Fig. 2C, 4,288 of the 8,421 models with at least one structural alignment hit are hypothesized to not be enzymes while the remaining 4,133 models pass the first stage of the enzyme annotation analysis. The side bar of panel C provides the breakdown of models that have been annotated as enzymes into the specific first-digit enzyme categories of the primary EC number annotations. Thirty-five models are seen to have statistical significance for hypothesizing that the modeled protein is an enzyme but do not get a primary EC number assigned because no consensus is achieved. The 4,133 models hypothesized to be enzymes represent 16.4% of all primary transcripts in the *S. divinum* proteome. This proportion of enzymes encoded in a species genome mirrors those of other eukaryotes, which, on average, have enzymes comprising 18% of their total proteome (Arakaki *et al.* 2006). These annotations and their respective aligned structures and statistics are made available for public use and further described in the SI.

Residue-level metadata is also considered to support the hypothesized EC number annotation. The feature and conservation count metrics highlight residues in the alignment hits’ structures that are associated with enzymatic activities, whether as the catalytically active residue or facilitating the binding of cofactors and substrates. A model’s residues that are strongly aligned with these feature residues from alignment hits are then hypothesized to have analogous functional relevance. Further consideration and comparison of EC number annotations developed from the structural alignment workflow and those initially reported in the JGI data portal for *S. divinum* is presented in Supplementary Section S3 in the SI.

### 3.3 Use of the structure-based EC annotation pipeline on proteins associated with stress response

Recent studies on *S. divinum* response to stress have identified several thousand proteins that are implicated in complex stress-response pathways (Healey *et al.* 2023). Ongoing investigations, including experiments on transcriptomic response to heat shock, are part of a campaign to understand these pathways. A set of 3,596 proteins were earmarked as being of special interest. Of these, 1,104 had sufficiently high predicted model scores to be passed to the annotation portion of the pipeline, 329 of which had at least one high quality alignment hits. Eighty-three of these proteins were matched with relative entropy and consensus scores sufficient for transfer of EC annotations according to thresholds defined by our method.

In the next sections, we focus specifically on three proteins from these studies where the results from our pipeline provide novel insights, demonstrating different applications of the method for uncovering clues about protein function. We also report results from gene expression experiments that confirm these proteins’ strong transcriptomic response to heat stress.

#### 3.3.1 Example 1: hypothesized cytochrome P450

The first example of the structural alignment for functional annotation workflow is the primary transcript, Sphmag13G047200. Expression studies have shown this protein to be significantly repressed in *S. divinum* plants under high temperatures, with –1.2 log2FC in Healey *et al.* (2023) and –1.6 in the current experimental results reported here.

The structural model of Sphmag13G047200 is depicted in Supplementary Fig.e S2A, colored by the residue-level pLDDT scores in a similar manner to that used in the AlphaFold DB. The structure has pTM and average pLDDT scores of 0.89 and 89, respectively, indicating a very high confidence model. Alignment against the PDB70 structural library returns 309 hits that surpass our average TM-score threshold of 0.7. In Supplementary Fig. S2B, the top ranked alignment is depicted where the model (purple) and 7CB9 (green; originating organism: *S. miltiorrhiza*) have an average TM-score of 0.88 and an aligned residue percent sequence identity of 33.5%.

Panels (C) and (D) of Supplementary Fig. S2 visualize the transfer of residue-level feature elements from the ensemble of alignment hits to the residues of the Sphmag13G047200 model. A single residue is highlighted by both the feature and conservation counts: Cys463 is aligned to feature elements a total of 226 times, with 224 of these instances having a cysteine residue conserved in the alignment hits. These 226 residue-level features are all categorized as “BINDING” with the associated ligand being “HEME”, as depicted in panel (B), where the Cys residue directly coordinates the Fe cation of the heme cofactor. Other residues in Sphmag13G047200 are highlighted by the feature counts metric, yet none to the extent of Cys463 and none with high conservation counts, indicating relative sequence and structural plasticity of the binding site residues in the set of 306 alignment hits, *excluding the essential Cys residue*.

**Figure 3.**
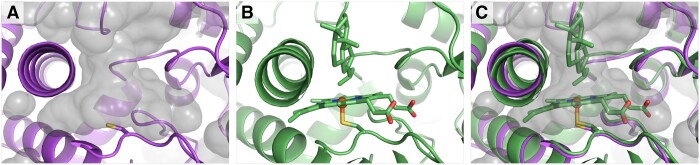
Putative heme-binding site in the model of Sphmag13G047200. (A) Protein-internal solvent accessible surface observed in the Sphmag13G047200 model (purple). The strongly conserved cysteine residue is shown with a licorice representation. (B) The 7CB9 crystal structure (green), focusing on the binding site of the heme cofactor and natural substrate (shown in licorice). (C) Structural alignment of the model with 7CB9, where both cofactor and substrate are well positioned within the volume of the accessible surface. No atomic clashes are observed between model atoms and the heme cofactor. Structural visualizations created using PyMOL (Schrödinger 2015).

Figure 3 depicts the close-up of the essential Cys residue and the putative heme-binding site in the Sphmag13G047200 model. Panel (A) depicts the solvent accessible surface around the cysteine residue in the model structure, where this surface represents a large, unoccupied volume within the core of the protein. From the same perspective, the crystal structure of 7CB9 is shown in panel (B). The heme cofactor and natural substrate, miltiradiene (a plant metabolite), are well resolved in the crystal structure. With a focus on this binding site, the alignment between 7CB9 and the Sphmag13G047200 model is depicted in panel (C). Both the heme cofactor and substrate from 7CB9 are positioned within the solvent accessible volume of the AlphaFold model. The model and crystal Cys residue sidechains overlap.

From the 309 alignment hits, 169 EC numbers were gathered where the prefixes 1.∗, 1.14.∗, and 1.14.14.∗ dominate the set with 151, 137, and 83 counts respectively; panel (E) of S2 depicts an EC number cloud to visualize these relative frequencies. The total relative entropy for these 169 EC numbers is 7.30 bits (*P*-value = 2e−234, *z*-score = 30), suggesting strong confidence in the sampled EC numbers, yet no single, full EC number achieves the strict majority consensus rule needed for annotating the model with a full label. Instead, the primary EC number annotation is 1.14.14.∗ as this is the first EC label that achieves the majority consensus; this is seen in the EC number logo shown in panel (E) of Supplementary Fig. S2. The 1.14.14.∗ EC number is a category for P450 heme-thiolate enzymes that function as monooxygenases on a broad range of possible substrates. The initial JGI annotation, developed from sequence alignment alone, suggests that this protein is homologous to TRANSPARENT TESTA 7 (TT7) in *A.thaliana*, which contains a cytochrome P450 domain and has flavonoid 3ʹ-hydroxylase (F3ʹH) activity (EC 1.14.14.82). TT7 is exceptionally well-characterized because it is a key component of the anthocyanin pathway used to biosynthesize flavonoid pigments that have profound impact on plant fitness and relevance to human health (He and Giusti 2010). However, assuming an identical substrate and function for this *S. divinum* protein may not be warranted, especially considering phylogenomic analyses found that seedless plants lack orthologs of many downstream genes in the anthocyanin biosynthetic pathway and suggested a paralogous relationship between Sphmag13G047200 and TT7/F3ʹH (Piatkowski *et al.* 2020). In fact, cytochrome P450 enzymes are a large superfamily of heme-containing monooxygenases with a broad range of substrates and chemical mechanisms (Alexander 2021). Therefore, we hypothesize that the more general 1.14.14.∗ EC label developed from the model’s structural alignment results is more accurate in regard to the current information we have for this protein. Additionally, the set of structural alignment hits for Sphmag13G047200 provides novel insights into the residue-level features that highlights the biochemically relevant residue hypothesized to coordinate a heme cofactor, increasing the supporting information for our annotation.

#### 3.3.2 Example 2: hypothesized pyridoxal $5^{\prime }$-phosphate synthase and homologous pseudoenzyme

Here, we consider two primary transcripts, Sphmag01G194900 and Sphmag02G160700, that encode proteins with respective sequence lengths of 312 and 311 residues and a sequence identity of 88.3%. Results from experiments measuring transcriptomic response to heat shock showed that Sphmag01G194900 and Sphmag02G160700 are both induced under heat stress, with a log2FC of 5.96 and 5.92, respectively. This degree of sequence similarity and stress–response leads to the assignment of these two proteins as paralogs originating from gene duplication (see Healey *et al.* (2023) for further discussion of the evolutionary history of *S. divinum*).

**Figure 4.**
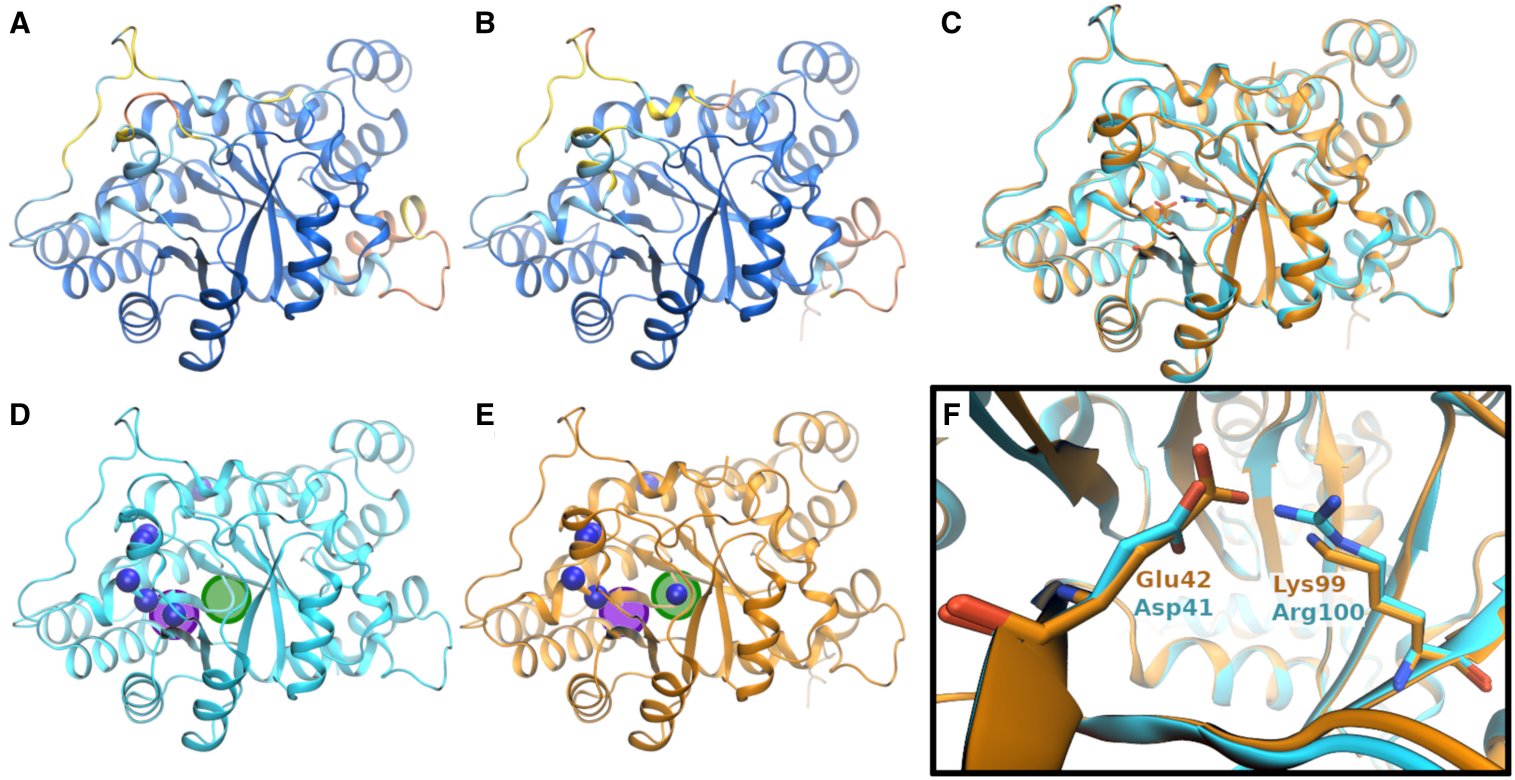
Structure model and residue-level feature results for Sphmag01G194900 and Sphmag02G160700. (A) and (B) Model quality visualizations of the two proteins, respectively. (C) Structural alignment of both models demonstrating strong structural homology between the two proteins (0.98 average TM-score and 88% sequence identity). (D) and (E) Conservation counts for both proteins, with the catalytic and binding residue positions highlighted in green and purple spheres, respectively. (F) Residue-level focus on these residues that form a salt bridge within the active site. The differences in residue type at these two structure positions is the supporting evidence for our hypotheses that Sphmag02G160700 is the cognate PLP synthase while Sphmag01G194900 is a pseudoenzyme. Structural visualizations created using VMD (Humphrey *et al.* 1996).

Figure 4A and B depicts the high-confidence structural models for these proteins. The pTM (average pLDDT) scores of Sphmag01G194900 and Sphmag02G160700 models are 0.89 (91) and 0.88 (90.), respectively. Alignment of the two models to one another, shown in Fig. 4C, returns an average TM-score of 0.98, indicating that the predicted models have nearly identical structures. When aligned using HHblits against the PDB70 sequence library, queries for both Sphmag01G194900 and Sphmag02G160700 return qualitatively identical results, differing only in ranking of the alignment hits. The sequence of 5LNR (chain D), a pyridoxal 5${}^{\prime }$-phosphate (PLP) synthase subunit (EC number: 4.3.3.6) from *A.thaliana*, is the first and third ranked sequence alignment hit for Sphmag01G194900 and Sphmag02G160700 with e-values (probability values) of $4.3\times 10^{-23}$ (99.7%) and $2.0\times 10^{-18}$ (99.5%), respectively. Structural alignment results for the two proteins mirror those of the sequence alignments; both proteins have identical structural alignment hits with slight variations in the quantitative metrics used for ranking. Only 14 structural alignment hits surpass the average TM-score cutoff with the top ranking hit for both proteins being chain G of 4WXY, a PLP synthase subunit from *G.kaustophilus*; these alignments, shown in Supplementary Figs S3 and S4, return the same average TM-score of 0.94 with aligned residue sequence identities of 62.8% and 60.1% for Sphmag01G194900 and Sphmag02G160700, respectively. Ten of the hits have an associated EC number, all of which are 4.3.3.6, resulting in a total relative entropy value of 9.5 bits (*P*-value = 7e−8; *z*-score = 5.3). Therefore, both proteins are labeled as enzymatic with 4.3.3.6 as the primary annotation hypothesis. Having the same alignment hits, the EC number logo and word cloud visualizations for these two proteins are identical (shown in Supplementary Fig. S5).

Differentiation of the two proteins is only obtained when the residue-level features are considered for this pair of proteins, depicted in Fig. 4 as well as in Supplementary Figs S3 and S4. Both proteins share six residues that are associated with binding site features, based on the hits’ UniProtKB metadata: Asp41 (Glu42), Gly172 (Gly171), Arg184 (Arg183), Gly233 (Gly232), Gly254 (Gly253), and Ser255 (Ser254) following the format of Sphmag01G194900 (Sphmag02G160700). One structure position in both models is labeled as an active site residue: Arg100 (Lys99). The conservation counts metric highlights the differences between the two proteins even further (Fig. 4D and E). The respective feature counts visualizations are provided in Supplementary Figs S3 and S4. For both panels, the catalytic residue position is highlighted with a green background while an adjacent binding site residue position is highlighted with purple. In panel (D), Sphmag01G194900 has Arg100 in the structural position associated with the catalytic residue, yet none of the hits’ active site features have an arginine residue in the analogous position. Conversely, Lys99 in Sphmag02G160700 is strongly conserved in the same set of alignment hits, seen in panel (E). A similar difference in conservation is seen in the binding site residue adjacent to the catalytic position: in Sphmag01G194900, an aspartate (Asp41) is highly conserved across the ten alignment hits while Sphmag02G160700 has a glutamate (Glu42) in this structure position. Figure 4F depicts the salt bridge that forms between the residues in the adjacent and catalytic structure positions. In Sphmag01G194900, the short side chain of Asp41 accommodates the larger guanidinium group of Arg100 while, in Sphmag02G160700, the reverse is seen for Glu42 and Lys99.

Consideration of the results of the full pipeline including residue-level analysis shows that while Sphmag01G194900 and Sphmag02G160700 proteins are strongly matched to PLP synthase subunits (EC number 4.3.3.6), the former lacks the catalytic Lys residue. Literature associated with PLP synthase subunits from *A.thaliana* indicates the presence of three paralogous subunits, two of which are cognate enzymes. The nonenzymatic protein in *A.thaliana*, labeled PDX1.2, has no innate activity in the PLP biosynthesis pathway and has been labeled as a pseudoenzyme (Tambasco-Studart *et al.* 2005, Moccand *et al.* 2014, Dell’Aglio *et al.* 2017, Robinson *et al.* 2019). In PDX1.2, the residue at the catalytic position is an Arg, just as it is in Sphmag01G194900. Therefore, the presence of Lys at the catalytic residue position in Sphmag01G194900 suggests that this PLP synthase paralog may be a pseudoenzyme with no innate enzymatic activity while Sphmag02G160700 is hypothesized to be a cognate pyridoxal 5${}^{\prime }$-phosphate synthase (EC number 4.3.3.6). This example highlights how residue-level details can be essential in distinguishing subtle differences in function even when global structure is nearly identical. Interestingly, in *A.thaliana*, the partnership between the PDX1.2 pseudoenzyme and the two cognate enzymes is seen to enhance the production of PLP (vitamin B_6_). Additionally, the expression of noncatalytic PDX1.2 is upregulated by heat and other stress conditions. We propose that a similar interplay between pseudoenzyme and cognate enzymes may be present for *S. divinum*.

## 4 Discussion and conclusions

From a methodology perspective, there are several caveats to be considered when using this workflow for functional annotation. First, the use of multiple tiers of cutoffs, acting on model quality and alignment quality aimed to limit the propagation of uncertainty into the final annotation hypotheses. The metrics and the numerical values used for cutoffs are suggestions and could be adjusted; these are discussed in more detail Supplementary Section S2 in the SI. Furthermore, there are multiple possible reasons why a high-confidence structural model may have few or no structural alignment hits. The limited representation of plant protein structures in the PDB propagates this bias into the PDB70 structural library used for this alignment analysis, potentially resulting in decreased numbers of alignment hits for *S. divinum* models. The original intent of the PDB70 *sequence* database was to be a representative set of *sequences* associated with structures in the PDB, with redundant sequences removed by applying a 70% identity filter. Transforming this sequence database into the PDB70 structural library does not fully appreciate the differences in 3D structures that redundant sequences may adopt. Structural alignment algorithms are especially sensitive to large scale structural differences such as shifts in domain orientations. If multiple conformers of a protein are present in the structural library, then it is possible that alignment hits may be found to only a subset of these.

In regard to the development of annotations, models with only a few structural alignment hits are inherently more difficult to annotate with the quantitative methods presented here, where small sample sizes increase the difficulty of obtaining statistical significance for annotating a protein with an EC number. The strict majority consensus rule applied to assigning a primary EC number annotation also leaves a small number of models in *S. divinum* with no annotation although these models are labeled as enzymes. In these instances of small-sample sizes or ambiguous annotations, further inspection of the structures and residue-level features may be required to determine the most accurate annotation.

The predicted proteome of *S. divinum*, the alignment results of this proteome against the annotated PDB70 structural library, the set of EC number annotations, and the accompanying residue-level feature information will aid investigation into *S. divinum*. Especially interesting are components of the proteome that contribute to antimicrobial products that are powerful antagonists to human pathogens (Opelt *et al.* 2007) and those proteins that contribute to the unique physiology supporting peatland carbon-sequestration under extreme environmental conditions. Beyond furthering studies of the *Sphagnum* complex, we anticipate that the datasets and software provided here will be used to drive analysis of other organism-scale structure prediction and annotation ventures.

## Supplementary Material

btad511_Supplementary_Data
