# Supplementary material for: Predicted structural proteome of Sphagnum divinum and proteome-scale annotation
Source: Bioinformatics. 2023 Aug 17;39(8):btad511. doi: 10.1093/bioinformatics/btad511 (PMC10463551; doi:10.1093/bioinformatics/btad511)
Supplement: btad511_Supplementary_Data [file btad511_supplementary_data.pdf]

Supplementary Information for
"Predicted structural proteome of Sphagnum divinum and proteome-scale annotation"

Russell B. Davidson, Mark Coletti, Mu Gao, Bryan Piatkowski, Avinash Sreedasyam, Farhan Quadir, David J. Weston, Jeremy Schmutz, Jianlin Cheng, Jeffrey Skolnick, Jerry M. Parks, and Ada Sedova

August 1, 2023

List of Figures

S1 Average pLDDT distributions of reference proteomes. 3
S2 Sphmag13G047200 structural visualization and EC number annotation. 4
S3 Sphmag01G194900 structural visualization. 4
S4 Sphmag02G160700 structural visualization. 5
S5 EC number annotation for both Sphmag01G194900 and Sphmag02G160700. 5
S6 PDB70 structural library UniProtKB overview and EC number counts. 8
S7 Effect of average TMscore cutoff value on number of alignment hits. 10
S8 Sphmag08G106500 structural visualization. 15
S9 Multiple sequence alignment and phylogenetic tree of Sphmag08G106500. 15
S10 Comparison of HHblits and US-align alignment metrics. 16
S11 Series of EC number logo figures for Sphmag13G047200. 17
S12 Timescaling of the SAFA workflow on OLCF Andes. 18
S13 Timescaling of the SAFA workflow on desktop computer resources. 19

Contents

1 Data Availability **6**

2 Materials and Methods - Continued **6**
2.1 AlphaFold Monomer v2 6
2.1.1 Model Quality Criteria 6
2.2 Development of the annotated PDB70 structure library 7
2.2.1 Gathering UniProtKB metadata. 7
2.2.2 EC number gathering. 8
2.3 Structural Alignment with US-align2 9
2.4 Quantifying EC number relative entropy for enzyme annotation 9
2.5 Visualization 11
2.6 Sequence alignments 12
2.7 Transcriptomic response to heat shock experiments 12
2.7.1 Plant material and experimental treatment. 12

2.7.2 RNA Extraction, Sequencing, and Analysis. 12

3 Consideration of initial JGI annotations 13

3.1 Annotation of Multifunctional Enzymes 13

4 Comparing structural alignment against sequence-based alignment 14

4.1 Large-scale comparison 15

5 Sequence alignment and phylogenetic tree of putative ribonuclease P proteins 17

6 Sphmag13G047200 - Hypothesized Cytochrome P450. 17

7 Dask-enabled workflow. 17

7.1 SAFA workflow on HPC 18

7.2 SAFA workflow on desktop computing resources 19

7.3 Discussion 20

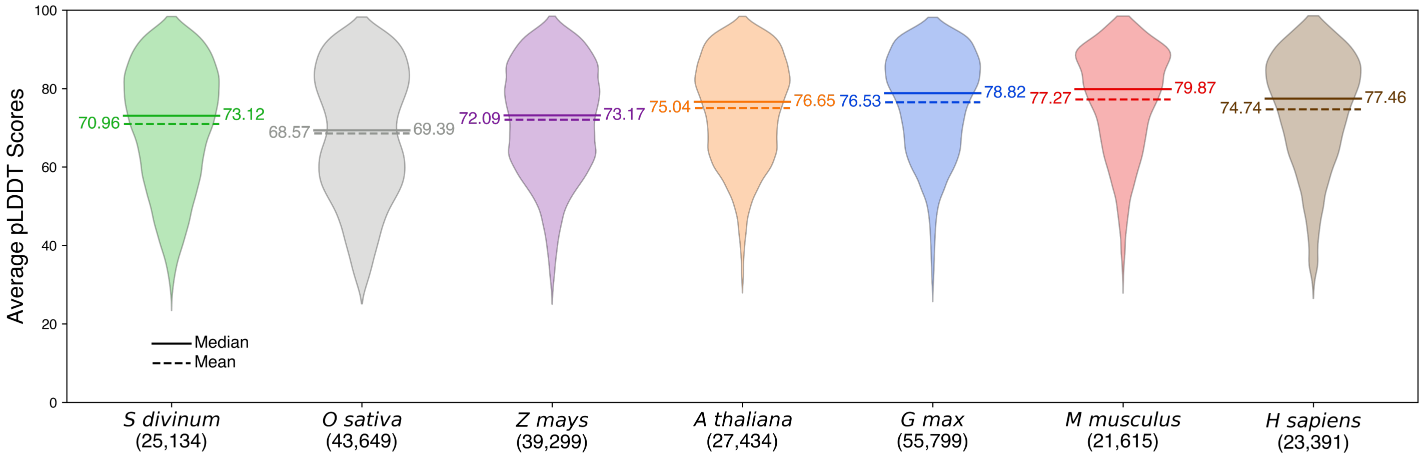

Figure S1: Average pLDDT distributions of *S. divinum* and other reference proteomes available through the AlphaFold DB. *O. sativa*, *Z. mays*, *A. thaliana*, and *G. max* represent the four major plant proteomes in the database while *M. musculus* and *H. sapiens* are a pair of proteomes originating from the animal domain. *G. max*, *M. musculus*, and *H. sapiens* distributions have the highest mean and median pLDDT values while *O. sativa* has the lowest for both statistics.

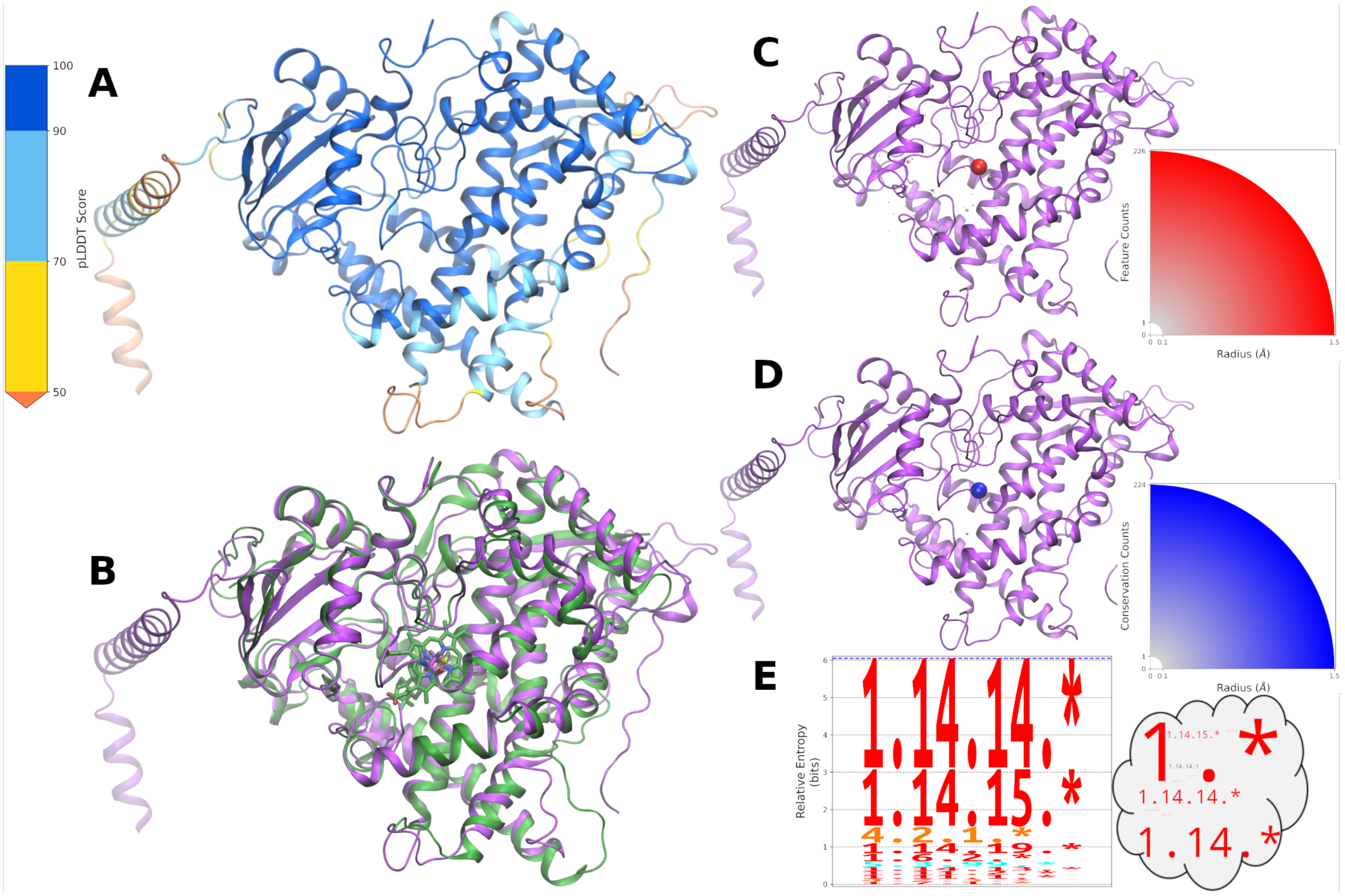

Figure S2: Structural inference and alignment results for Sphmag13G047200, a hypothesized cytochrome P450. (A) Model quality visualization with residues colored by pLDDT scores. (B) Top structural alignment visualization between the model (purple) and 7CB9 (green). Structural visualization of the (C) feature and (D) conservation counts of residues in the 309 structural alignment hits, both highlighting the heme-binding Cys residue. (E) EC number logo and cloud representations of the alignment results. The consensus EC number in the logo figure suggests an annotation of 1.14.14.∗. Structural visualizations created using VMD [1].

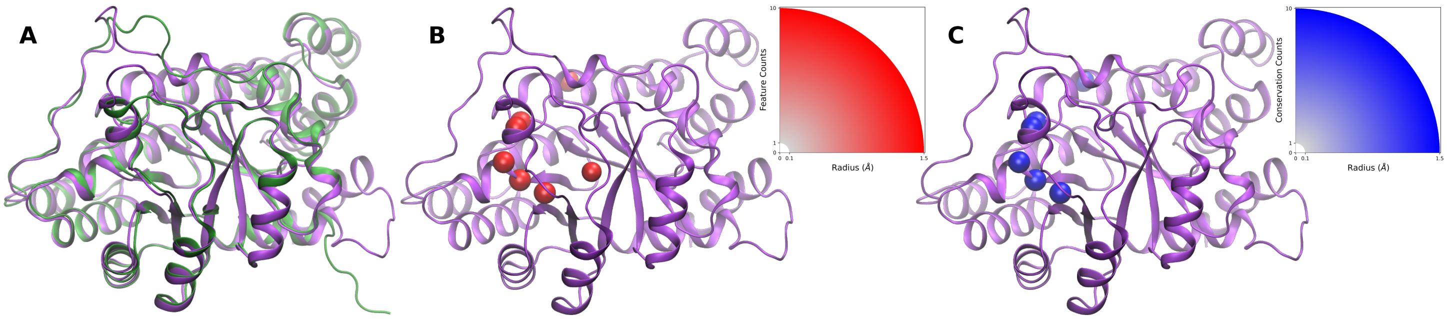

Figure S3: Structural alignment results for the Sphmag01G194900 primary transcript. (A) Alignment between the model (orange) and PDB 4WXY, chain G (green; a pyridoxal 5′-phosphate synthase from *G. kaustophilus*). The average TM-score of this alignment is 0.94 with a aligned sequence identity of 62.8%. (B) Structural visualization of the feature counts metric used to highlight the seven residues in the model that are aligned to PDB70 structure residues annotated as active or binding site. (C) Structural visualization of the conservation counts metric, presented in the manuscript.

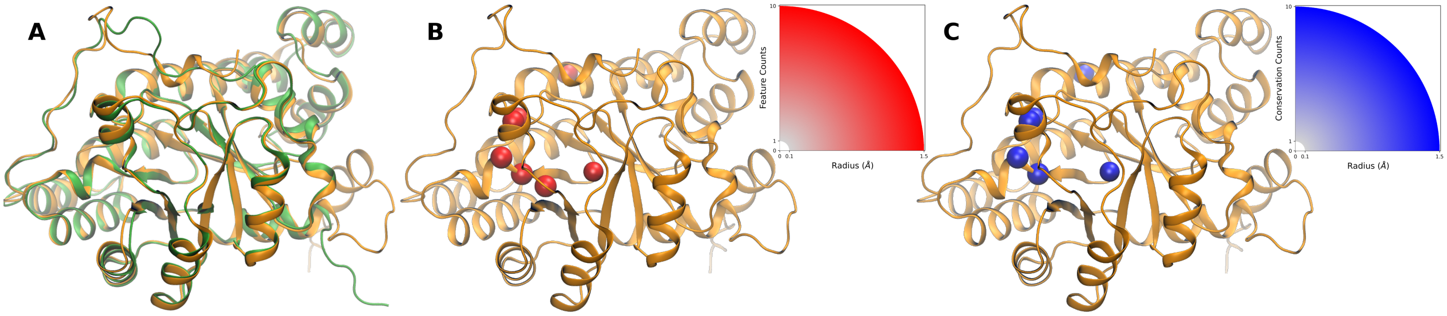

Figure S4: Structural alignment results for the Sphmag02G160700 primary transcript. (A) Alignment between the model (orange) and PDB 4WXY, chain G (green; a pyridoxal 5′-phosphate synthase from *G. kaustophilus*). The average TM-score of this alignment is 0.94 with a aligned sequence identity of 60.1%. (B) Structural visualization of the feature counts metric used to highlight the seven residues in the model that are aligned to PDB70 structure residues annotated as active or binding site. (C) Structural visualization of the conservation counts metric, presented in the manuscript.

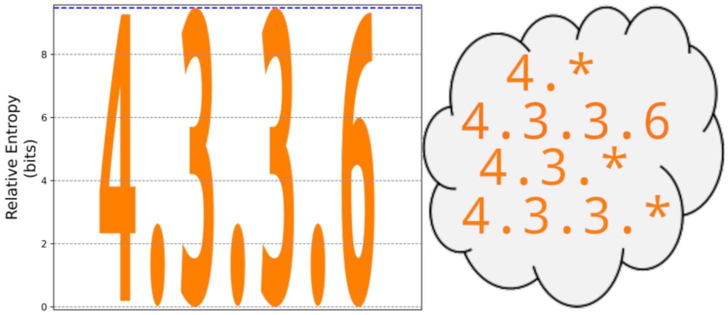

Figure S5: Sphmag01G194900 and Sphmag02G160700 protein models align strongly to the same set of 14 PDB70 structures. 10 EC numbers are gathered from these alignment hits, which produce the EC number logo and wordcloud presented here.

1 Data Availability

For each protein in the *S. divinum* proteome, the highest pTM scoring predicted structural model is deposited in the ModelArchive repository at `https://modelarchive.org/doi/10.5452/ma-ornl-sphdiv`. The sequence and structural alignment results are available through through Constellation, the Oak Ridge Leadership Computing Facility (OLCF) DOI-based science network for supercomputing data, at `https://doi.org/10.13139/ORNLNCCS/1973885`; this data repository also houses all five structural models output from AlphaFold. Finally, the PDB70 structural library and associated metadata is available at `https://doi.org/10.5281/zenodo.7953087`. The code for the structural alignment and annotation pipelines is available at `https://github.com/BSDExabio/SAFA`, along with Jupyter notebooks for demonstration. Finally, expression data for specific heat-stress response proteins are publicly available through the NCBI under BioProject PRJNA962985 and SRA accessions SRR24348249 through SRR24348254.

The *S. divinum* genome, NCBI Taxonomy ID 128215, is available through the JGI Phytozome data portal as the file `Smagellanicum_521_v1.1.cds_primaryTranscriptOnly.fa.gz`.

2 Materials and Methods - Continued

2.1 AlphaFold Monomer v2

Inference of structural models using AlphaFold Monomer v2 [2] was performed using a massively parallel implementation, as described previously [3]. Briefly, preprocessing of each *S. divinum* protein sequence was performed on OLCF Andes. Multiple sequence alignments (MSAs) and structural templates were calculated using Kalign [4], HMMER, and HHSuite [5] using the MGnify [6], UniRef90, UniClust30 [7], PDB70 [5], PDB, and reduced BFD sequence libraries. A version of AlphaFold v2.0 (`https://github.com/deepmind/alphafold`) was modified for deployment on the OLCF Summit supercomputer and run with a dynamic recycling procedure. Specifically, recycles were performed while the difference in between-recycle residue contact distograms was above a cutoff of 0.5 Å; a maximum of 20 recycles was performed for small proteins and scaled down as the protein residue length increased. Next, clash reduction refinement was performed using the OpenMM molecular dynamics program (v. 7.6) [8] and the AMBER ff99SB force field [9]. Top models were chosen using the best predicted template modeling score (pTM) and were used for the remainder of the structural alignment for functional annotation (SAFA) workflow.

2.1.1 Model Quality Criteria

As stated in the manuscript, the model quality criteria is a pTM score greater than 0.7 or a predicted Local Distance Difference Test (pLDDT) score greater than 70. A brief reasoning for this is provided in section 3.1 of the manuscript. In more detail, the pLDDT is a per-residue, local model quality metric that is then averaged to describe the global structural model quality, termed the average pLDDT score in the manuscript. In instances where a high-confidence globular structure is inferred but is plagued by low-confidence termini or loop regions, the average pLDDT is describing a multi-modal distribution of per-residue pLDDT scores, resulting in a lower average pLDDT than might be appropriate for the globular domain – the domain of interest in this work. The pTM is a predictive metric that was shown to correlate with TM-score values from structural alignment analyses in [2]. Since TM-score describes the quality of global structural alignment, the pTM is used as a global model quality metric and is not as sensitive to local effects such as poorly modeled loop regions. So, if a structural model has a significant proportion of disordered residues but a high confidence globular domain, the pTM metric is more likely to be the model's redeeming quantitative value. Therefore, we propose that both pTM and average pLDDT metrics should be used when considering the overall quality of structural models produced from AlphaFold.

The specific cutoff values for the criteria was made arbitrarily; we followed the suggested guidelines reported in the AlphaFold Protein Structure Database (AFDB) FAQ. Unfortunately, the pTM score for models in the AlphaFold

Protein Structure Database are not available and the metric is not discussed. Users of the SAFA workflow can define their own cutoffs, if they wish.

2.2 Development of the annotated PDB70 structure library

The PDB70 sequence database (dated 2022-03-13) was used as the origin for the PDB70 structural library, reported here. The intent of the PDB70 sequence database is to be "a representative set of sequences from the PDB, filtered with a maximum pairwise sequence identity of 70%" [5]. The sequence identity filtering minimizes the number of redundant sequence entries, which, as an initial assumption, will also likely minimize redundant structures. This assumption is flawed to some extent, for numerous reasons, such as structural variety in domain-domain orientations, structural changes between apo- and holo-state protein conformations, or structural heterogeneity in poorly-resolved or highly dynamic regions of proteins. In these ways, the structure library developed from the PDB70 sequence database is likely to under-represent the structure space of the full PDB database; development of a more representative structure database is beyond the scope of this paper.

There are 92,111 sequence entries in the PDB70 sequence database; 51 of the associated structures were unavailable to download when preparing the structure library on March 27, 2023. A further 1,035 structures were removed from the library due to being too short (< 30 residues in length) or being too low-resolution ($< 75\%$ of the encoding sequence is structurally resolved). This leaves a total of 91,032 structures in the the PDB70 structural library used in the structural alignments for this manuscript. These 91,032 structures originate from 69,782 unique PDB accession IDs, many of which are representations of heteromeric protein complexes. To gather the structures, the **mmtf** and **MDAnalysis** [10, 11] Python modules are used.

2.2.1 Gathering UniProtKB metadata.

For each entry in the PDB70 structural library, the PDB accession ID and chain ID were used to gather the UniProtKB [12] accession IDs, when available. To do so, the GraphQL-based API for accessing the RCSB PDB was used. If a UniProtKB accession ID is available for the protein structure, then the UniProtKB REST API was used to pull the corresponding metadata flat file. Each flat file was parsed to gather most of the associated metadata, excluding the literature references for the protein. For the work reported here, focus is given to the enzyme commission (EC) number(s) listed in the "DE" lines in the flat file as well as the residue-level feature elements (the "FT" lines).

Due to the potential for ambiguity between PDB structure residue numbering and UniProt sequence indexes, a BlastP [13] alignment was performed (via the **Biopython** module) to map the two sequences. In this way, correct residue-level metadata listed in the UniProtKB flat file was transferred to the associated PDB structural representation. The results from this alignment analysis mirrors the strategy used by SIFTS [14, 15], provided by the Protein Data Bank in Europe (PDBe) in collaboration with the UniProtKB.

Finally, it is important to emphasize that residue-level features reported in a UniProtKB flat file are not guaranteed to specify any connection to other metadata fields or evidence sources. And even when provided, evidence sources often point to the automated analysis or originating article where the feature was first identified and/or Evidence Ontology IDs. For "BINDING" and "ACT_SITE" features, there may be a note field that gives a description of the function associated with the feature. Due to this indirect connection between residue-level information and classification ontologies (e.g. EC numbers) in the UniProtKB flat files, feature elements are not used for the development of annotation hypotheses. Rather, this information is gathered as supporting information for hypotheses developed during the SAFA workflow (see Section 2.4).

For the current version of the PBD70 structural library, structures and metadata were downloaded on March 27, 2023.

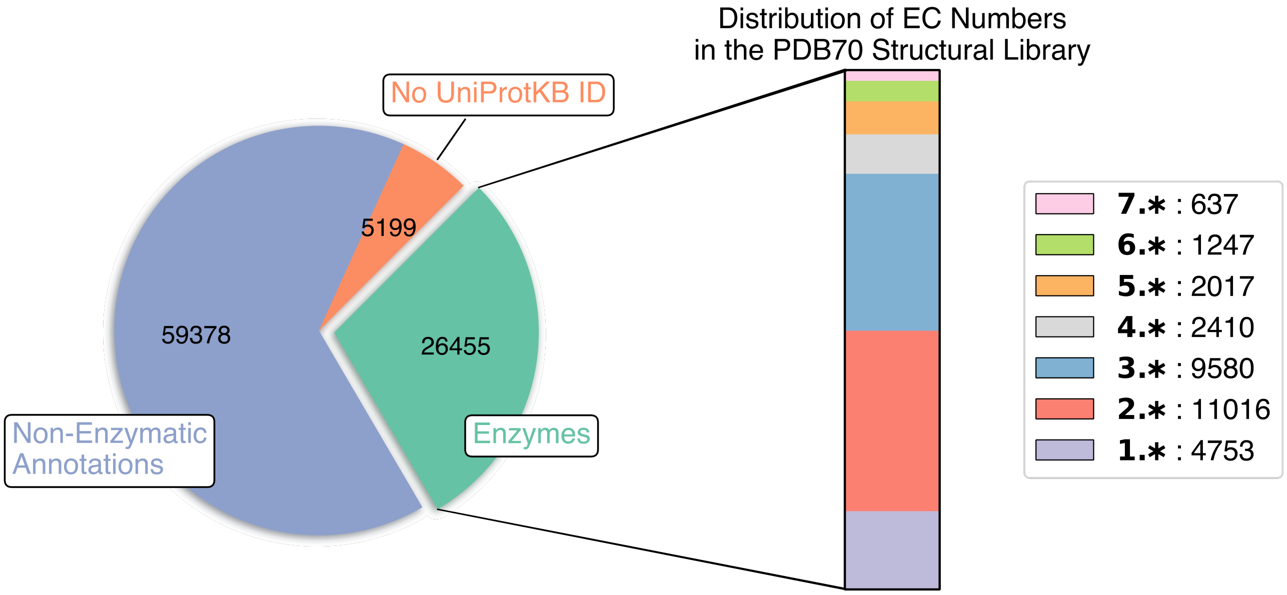

Figure S6: Distribution of annotated structures in the PDB70 structural library. (Left) 5,199 structures have no associated UniProtKB Accession ID, so have no annotation metadata. The remaining structures' UniProtKB flat files have been parsed for a large variety of metadata. 26,455 of these structures have an active EC number listed in their metadata. (Right) Stacked bar plot of first digit EC numbers gathered from the structures in the PDB70 structural library. A total of 31,660 active EC numbers are gathered from the structures, which are distributed across the seven classes of enzymes as shown.

2.2.2 EC number gathering.

Figure S6 shows the break down of the PDB70 structural library that do not have UniProtKB accession IDs as well as those that have an associated UniProtKB flat file, whether the annotations indicate the structure is enzymatic or not. Only 5,199 structures lack an associated UniProtKB accession ID; we have very little metadata beyond the structural model for these PDB70 structures. From the 85,833 structures with UniProtKB accession IDs, 26,455 structures are annotated with at least one active EC number, where "active" EC numbers are defined as those listed in the BRENDA database (`https://www.brenda-enzymes.org/all_enzymes.php`) [16, 17]. BRENDA is a high-quality, human-curated database for enzyme categorizations and functional details. If a UniProtKB flat file contains an "inactive" EC number annotation, whether the number has been deleted or transferred, the BRENDA list is used to identify an alternate, "active" EC number that can be used instead. For example, the EC number 1.8.6.1 (nitrate-ester reductase) is labeled as "deleted" in the BRENDA database, where entries previously annotated with this number have been transferred to 2.5.1.18 (glutathione transferase). If no alternate number is listed, then no EC number is gathered from the associated UniProtKB metadata; an example of such an instance is 1.2.1.7 (benzaldehyde dehydrogenase) that is listed as deleted with no replacement nor transferal to some other EC number. It is beyond the scope of the development of the structure library to determine if the reclassification of a structure's inactive EC number is appropriate. This strategy of finding a replacement for defunct EC numbers is employed to avoid the potential loss of enzymatic annotation information that arises when UniProtKB metadata flat files are not frequently updated to represent the most accurate categorizations or annotation information available; this represents a miscommunication between UniProtKB entries and actively maintained EC number databases such as BRENDA or Expasy.

In addition to defunct EC numbers, there are 6,621 structures in the PDB70 structural library associated with UniProtKB flat files that have preliminary (e.g., 4.2.2.n1) or incomplete EC numbers (e.g., 1.14.14.-). These instances of non-active annotations are not considered for the hypothesis development analyses but still may provide further insights into enzymatic functional annotations when present in a model's alignment hits' metadata (see Section S6 below).

As presented above, there are 26,455 structures in the PDB70 structural library annotated with at least one active EC number. In this population, there are 2,628 structures that are multifunctional, having more than one EC number annotation. For these multifunctional enzymes, each EC number is counted independently when

considered in annotation hypothesis development (see Section S2.4). The total number of active EC numbers in the PDB70 structural library is 31,660. The distribution of active EC numbers observed in the PDB70 structural library across the 7 broad categories for enzymatic activity is shown in Figure S6.

2.3 Structural Alignment with US-align2

The structural alignment calculations are performed using US-align2 [18] and specifically the semi-non-sequential (sNS) alignment algorithm [19]. Non-sequential alignment algorithms, such as sNS, are capable of aligning protein structures that have similar 3D topologies but drastically different linear sequences [20], which may be the case for convergent evolution of structural motifs across kingdoms and phyla. Since structures in the PDB70 structural library are gathered from a diverse set of originating organisms, the sNS alignment algorithm was deemed sufficient to perform billions of alignment calculations due to the trade-offs of computational efficiency and alignment quality. A workflow using the Dask parallel library for Python [21] has been implemented to automate and scale the large number of alignment calculations to leadership-scale HPC allocations. See Section S7 for more information about the workflow.

US-align2 reports several quantitative metrics that describe the quality of structural alignment. The two template modeling scores (TM-scores) [22] quantify structural similarity of the two aligned proteins, where each TM-score is normalized to a bounded range between (0,1]. Alignments against the PDB70 structural library are full-chain comparisons; the intent is finding structural analogs rather than focusing on domain-specific details. Therefore, the average of the two TM-score values is used as the quantitative metric for ranking alignment results and guiding further consideration.

A conservative average TM-score cutoff of greater than 0.7 is applied when describing “good” alignments, or “hits”, to ensure consideration of only high-quality structural alignments. Figure S7 depicts the histograms of number of *S. divinum* models versus the number of structural alignment hits as the average TM-score cutoff is changed from 0.5 to 0.9. As one might expect, increasing the cutoff to larger values results in fewer alignment hits for the population of structural models. As discussed in the manuscript, models with few alignment hits represent instances of small sampling that have difficulty when developing enzymatic annotations. Additionally, the visual analysis of structural alignments ranging in average TM-score values supports a more conservation, larger-valued cutoff; the qualities of structural alignments are hugely different between alignment pairs that have average TM-score values of 0.5 and 0.9. Therefore, we utilize the > 0.7 average TM-score cutoff for the work reported here.

In addition to gathering the quantitative metrics output from US-align2, the alignment mapping of residues in query and target structures is also kept for further analysis. This information is essential when considering the transfer of residue-level metadata from alignment hits to the structural model (i.e. feature and conservation counts discussed in the manuscript). The translational and rotational matrix to align each alignment hit to the structural model is saved to storage so that the alignment can be recreated for further analysis and visualization.

2.4 Quantifying EC number relative entropy for enzyme annotation

As presented in the manuscript, D_{KL} is a statistical distance between the observed and background probability distributions, which in this application are the probabilities of EC numbers in alignment hits' metadata and the background probabilities of all active EC numbers in the PDB70 structural library. When a model's alignment hits have a “narrow” distribution of EC numbers, localized on one or a few labels, D_{KL} will report a “large” distance between the two distributions that quantifies the information gained about the model's enzymatic annotation.

To avoid numerical inaccuracies when calculating D_{KL}, a pseudocount is added to each EC number in X before P is calculated. This pseudocount value is variable based on whether considering full EC numbers or prefixes, using the equation:

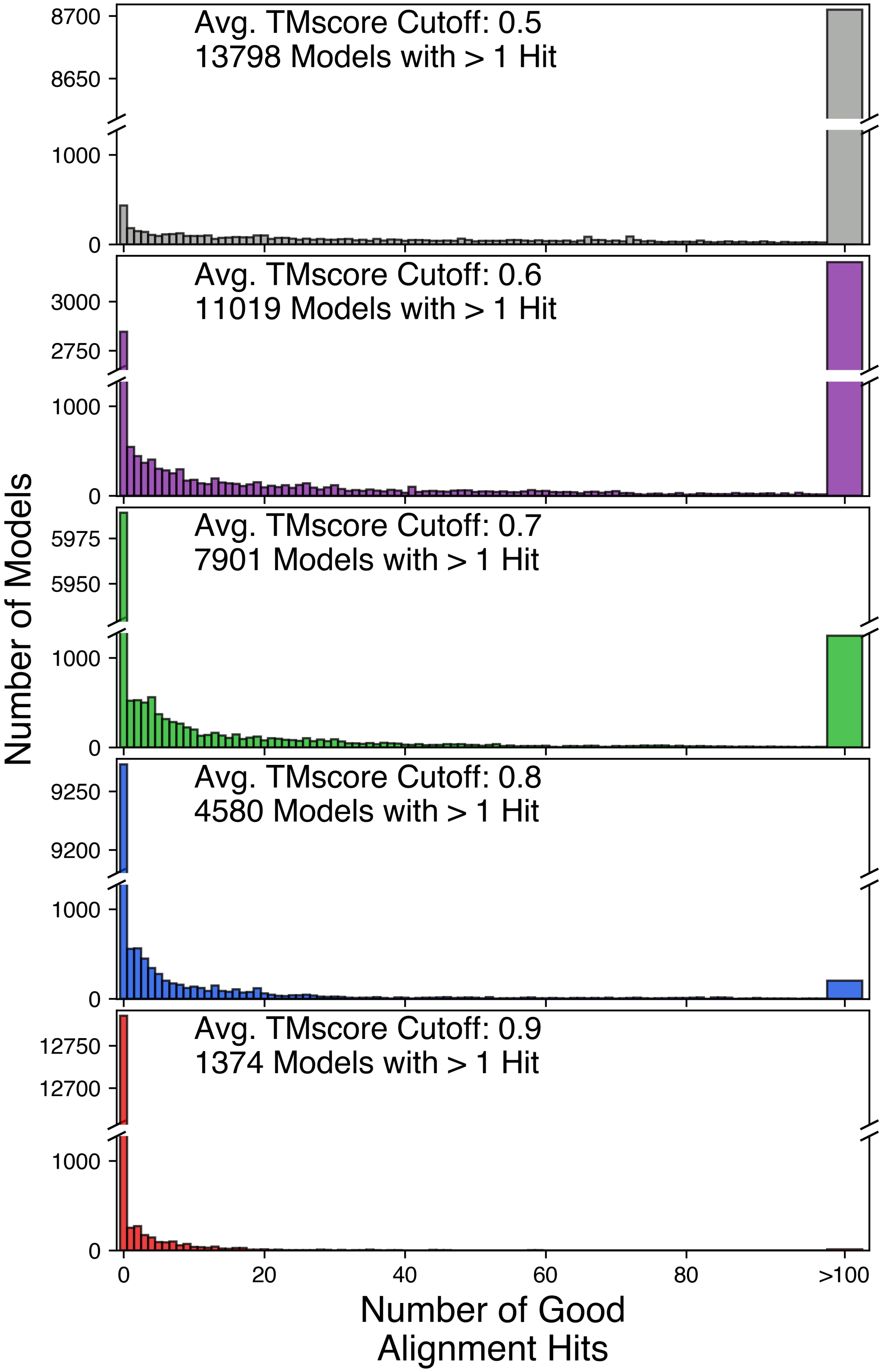

Figure S7: Histograms of number of models with a number of structural alignment hits as a function of the average TM-score cutoff used to define "good" alignment hits. From top to bottom, the average TM-score cutoff is 0.5, 0.6, 0.7, 0.8, and 0.9. As the cutoff becomes more stringent, the number of models with zero alignment hits increases, indicating a loss in predictive power. Using too low of a cutoff results in a loss of accuracy and precision, where most models are observed to have 100 or more alignment hits.

$$p = \frac{1}{n_{IDs}}, \quad \begin{cases} \text{first digit (e.g., 1.*)}, \; n_{IDs} = 7 \\ \text{second digit (e.g., 1.1.*)}, \; n_{IDs} = 74 \\ \text{third digit (e.g., 1.1.1.*)}, \; n_{IDs} = 243 \\ \text{full EC numbers (e.g., 1.1.1.1)}, \; n_{IDs} = 3292 \end{cases} \tag{1}$$

where p is the pseudocount value for the respective completeness of the EC labels and n_{ID} is the total number of unique EC numbers represented in the PDB70 structural library.

The numerical value of the relative entropy is dependent on the number of observed ECs in the model's alignment hits (n_{obs}) as well as those numbers' uniqueness in the background distribution. Therefore, it is intractable to use a uniform cutoff on the D_{KL} distance as the quantitative criterion for declaring whether a model is annotated or not. Instead, bootstrapping is used to resample the D_{KL} space. 1 million iterations of randomly pulling n_{obs} EC numbers from the background distribution is performed; the total relative entropy is calculated for each iteration and represents a possible D_{KL} value if n_{obs} EC numbers were to be randomly chosen. For each n_{obs} value, the associated bootstrapped D_{KL} distribution describes the expected range of values when the set of ECs are randomly drawn from the background list of EC numbers. If the model's observed relative entropy value is greater than the 95th percentile of the bootstrapped D_{KL} distribution, then the model is hypothesized as *being an enzyme* and is labeled as such. For large n_{obs} values, this is equivalent to performing a right-tailed p-value hypothesis test with a significance level of 0.05.

Once classified as an enzyme, a model is annotated with a primary EC number as discussed in the manuscript. Specifically, all EC numbers' component to the total relative entropy value (the term within the summation in Eqn. 1 in the manuscript) are considered. Starting with full EC numbers, if one EC number's component represents 50% or more of the total relative entropy value, then the protein is annotated with that EC number. If no full EC number passes this consensus rule, then the annotation decrements back into the EC number prefixes from three- to two- to one-digit labels until a consensus is obtained. If no consensus is obtained for even the first digit EC number, the model is annotated as “no consensus." Whether annotated with a primary EC number or not, the ensemble of observed EC numbers should still be considered; disparate enzyme categories may be associated with structurally similar proteins and cofactors. Furthermore, annotation with a primary EC number is not mutually exclusive with other EC numbers being relevant to the modeled protein. Although rare, multifunctional enzymes are expected to be present in the *S. divinum* proteome. Currently, these are undetected using the majority consensus rule for primary EC number annotation. If a protein is seen to have multiple well-sampled EC numbers in its respective alignment results, then the “primary” EC number may not be the only relevant number to be reported in the annotation. Numerous EC numbers can be considered for a model's annotation if the respective relative entropy components are equivalently large or statistically significant from the background distribution. See Section S3.1 for further consideration of multifunctional enzymes in the *S. divinum* proteome.

2.5 Visualization

To visualize the EC number results, two representations are produced: a simple word cloud and a relative entropy plot. The word cloud represents all observed full, four-digit EC numbers as well as the EC number prefixes. The font sizes of EC numbers are scaled by the respective frequency: the larger the EC label in the cloud, the more common that number in the alignment hits' metadata. Sparsely filled word clouds are suggestive of a consensus in EC numbers. The relative entropy plot is a graphical depiction that mirrors those of sequence logos often used to visualize the conservation of sequence positions in a multiple sequence alignment [23, 24]. Specifically, a model's EC number logo is the stack of EC numbers observed in the alignment hits' metadata, with the font size of each label set based on its relative frequency. Each EC number within the stack is colored by its first digit to differentiate and highlight the general enzymatic activity suggested from that label. The total height of the stack is calculated using the Kullback-Leibler divergence, D_{KL}, defined in the manuscript. A separate logo figure is made for full EC numbers and prefixes. Word clouds, EC number logos, and other graphs were generated using **word_cloud**, **matplotlib** [25], and **pillow** [26] Python modules.

Structural visualization of predicted models and alignment hits are paramount when supporting the hypotheses proposed from the aggregated EC numbers and feature information. Structural visualization figures were made using VMD [1] except where stated otherwise.

2.6 Sequence alignments

For direct comparison of sequence alignment and structural alignment against the PDB70 structural library, HHblits [5, 27] alignment was performed for each of the stress-response-related proteins in *S. divinum* against the PDB70 sequence database [5] (same version number as used for the structural database – 2022-03-13). For each protein associated with the heat-stress response, the below HHblits command was used:

```
hhblits -cpu 4 -diff inf -n 3 -e 0.001 -cov 60 -id 99 \
        -i /path/to/fasta \
        -d /path/to/pdb70_database \
        -oa3m /path/to/output/a3m
```

The `hhr_reader.py` script from `https://github.com/soedinglab/hh-suite/blob/master/scripts/hh_reader.py` was used to parse the hhr files for sequence alignment results.

2.7 Transcriptomic response to heat shock experiments

New gene expression analysis experiments were performed to validate the transcriptomic responses of Sphmag01G194900, Sphmag02G160700, and Sphmag13G047200 to heat shock, which were first reported in [28]. These three proteins are focused on here as examples of how our structural bioinformatics pipeline can help elucidate the functions of important proteins uncovered by such expression studies.

2.7.1 Plant material and experimental treatment.

Material of *Sphagnum divinum* was collected from the SPRUCE site in the Marcell Experimental Forest north of Grand Rapids, MN (47.506° N, 93.453° W). The mosses were grown in 1020 trays on Fafard 52 media (Sun Gro Horticulture, Agawam, MA, USA) under 25 °C (day)/22 °C (night) $\pm$ 3 °C conditions in the greenhouse. To quantify the plant's transcriptomic response to heat shock, we transferred plants to Conviron CMP 3244 growth chambers set to 23 °C (control) and 45 °C (treatment). As peat mosses can hold large amounts of water [29], one tray containing 5 L of distilled water was placed into each growth chamber three hours prior to transferring the plants so that high temperatures were experienced more rapidly in the treatment samples and the effects of desiccation were mitigated. Two hours after transfer to the growth chambers, three samples were collected for each treatment condition with each sample comprised of 5 bulked plants. Samples were flash frozen in liquid nitrogen and placed into a -80 °C freezer until nucleic acid extraction.

2.7.2 RNA Extraction, Sequencing, and Analysis.

Frozen plant tissue was ground using a 2010 Geno/Grinder Automated Tissue Homogenizer (SPEX, Metuchen, NJ, USA). Tissue was lysed with CTAB buffer and nucleic acids were extracted using chloroform-isoamyl alcohol and the Sigma Spectrum Total Plant RNA extraction kit (Sigma-Aldrich, Burlington, MA, USA). DNA digestion was performed using the Sigma on-column DNase I digestion kit (Sigma-Aldrich, Burlington, MA, USA). RNA library preparation and sequencing was conducted at Azenta Life Sciences (South Plainfield, NJ, USA). RNA samples were quantified using a Qubit 2.0 Fluorometer (Life Technologies, Carlsbad, CA, USA) and RNA integrity was checked using an Agilent TapeStation 4200 (Agilent Technologies, Palo Alto, CA, USA). RNA sequencing libraries were prepared using the NEBNext Ultra II RNA Library Prep Kit (NEB, Ipswich, MA, USA) according to the manufacturer's instructions. The mRNAs were initially enriched with Oligo d(T) beads, fragmented for 15 minutes at 94 °C, and used for cDNA synthesis. cDNA fragments were end repaired and adenylated at

3' ends. Universal adapters were then ligated to cDNA fragments, followed by index addition and library enrichment by PCR. Libraries were validated on the Agilent TapeStation and quantified using the Qubit 2.0 Fluorometer and quantitative PCR (KAPA Biosystems, Wilmington, MA, USA). Sequencing was performed on an Illumina HiSeq 4000 using a 2x150 paired-end configuration. Quality control was performed for the sequencing reads with FastQC v0.12.1 (`https://www.bioinformatics.babraham.ac.uk/projects/fastqc/`). TrimGalore v0.6.10 (`https://zenodo.org/badge/latestdoi/62039322`) was used to perform quality and adapter trimming with default settings but removing reads containing at least four 'N' bases. Gene expression was quantified with salmon v1.10.1 [30] using the v1.1 annotation of the *S. divinum* reference genome [28], `https://phytozome-next.jgi.doe.gov/info/Smagellanicum_v1_1`. Differential gene expression analysis was performed using DESeq2 v1.36.0 [31] with a statistical significance level of $P < 0.05$ after controlling the false discovery rate with the Benjamini-Hochberg procedure and a 0.584 log fold change threshold.

3 Consideration of initial JGI annotations

During the initial genome annotation pipeline [28], a BlastP [13] sequence alignment was performed between the predicted *S. divinum* primary transcripts and the proteomes of *A. thaliana*, *O. sativa*, and *C. reinhardtii*. These sequence alignment results were used to propose initial annotations for the primary transcripts, including EC numbers, GO, KEGG, PFAM and other metadata terms. These initial annotations are reported in the annotation information file available in the JGI Phytozome catalog (`Smagellanicum_521_v1.1annotation_info.txt`). From this automated annotation pipeline, all but 3,414 *S.divinum* proteins have been categorized with at least one annotation term, although the confidence of the BlastP alignments are not reported. For these proteins with no functional annotation information, 490 have high-quality structure models predicted by AlphaFold 2, from which 134 models have one or more structural alignment hit in the PDB70 structural library. This subset of unannotated proteins represents an important focus area where the structural model and alignment results can provide novel information. The remaining portion of the *S. divinum* proteome is also ripe for study with our structural alignment workflow. As presented in the next section, sequence-alignment methods may result in incomplete or inaccurate annotation transfer.

In regards to enzyme annotations, 1,431 of the EC number annotations developed by our SAFA pipeline are exact matches to the associated metadata reported in the JGI file. Another 1,628 models' annotations are partial matches to the JGI versions, where agreement between EC numbers is down to the first, second, or third digit of the classifier. Wholly different EC numbers are observed in 245 models' JGI and structural alignment annotations, although the pairs of EC numbers are often synonymous or are associated with the same biochemical pathways. Finally, 794 of the EC number annotations developed from our pipeline are completely novel: the associated JGI annotation information for these proteins does not include an EC number.

3.1 Annotation of Multifunctional Enzymes

In the JGI annotation file, there are 514 *S. divinum* primary transcripts that are annotated with multiple EC numbers: 288, 107, 38, and 81 proteins have multiple EC numbers that vary by the fourth, third, second, or first digits, respectively. 258 of these JGI-annotated multifunctional enzymes are not annotated in the SAFA workflow; these instances require further inspection to determine the validity of both JGI and SAFA analyses. The remaining 256 proteins have varying levels of agreement between the multiple JGI-reported EC number hypotheses and the EC numbers observed in the SAFA results, naive of the primary EC number annotation derived from the relative entropy analysis. From this subset of proteins, the SAFA results for 148 *S. divinum* primary transcripts have structural alignment hits that represent all JGI-annotated EC numbers for the respective protein, although relative entropy values associated with these EC numbers can range in significance.

For example, the Sphmag05G086600 primary transcript has eight structural alignment hits, all of which are labeled in the associated UniProt flat files with both 2.7.1.105 and 3.1.3.46 EC numbers; the average TMscore

values for these alignments are all ∼0.71. Due to a slightly smaller population of structures with the 2.7.1.105 annotation in the PDB70 structural library, the relative entropy consensus rule assigns the primary annotation for Sphmag05G086600 as 2.7.1.105 (51% of the total relative entropy) while 3.1.3.46 represents 49% of the total relative entropy. Both EC numbers have significant relative entropy components and this primary transcript is hypothesized as multifunctional, playing an important role in the metabolism of fructose and mannose. This SAFA-derived annotation of Sphmag05G086600 as a multifunctional enzyme agrees with the EC numbers originating from the JGI file.

Conversely, Sphmag15G018300 is annotated as 3.7.1.8 and 3.7.1.17 in the JGI annotation file. Individually, these EC numbers represent 7% and 3% of the total relative entropy value from the SAFA analysis. The model's 195 structural alignment hits have a diverse set of EC numbers from the subtree of hydrolases (3.*), resulting in the primary EC number annotation being 3.∗. Although the JGI-annotated EC numbers are observed in the alignment hits' metadata, the SAFA annotation suggests that the protein model has structural similarities with a broad range of hydrolase enzymes; there is insufficient confidence to assign annotations at the full EC number level.

Finally, there are 108 instances where a primary transcript's SAFA results lack sampling of one or more JGI-annotated EC numbers. For Sphmag03G067200, seven structural alignment hits resulted in a unanimous annotation as 4.6.1.13 with no other EC number being represented. The JGI EC number annotation is 4.6.1.13 and 3.1.4.11. In the PDB70 structural library, there are 35 examples of 3.1.4.11 structures, none of which strongly align to the protein model. Further investigation is required to determine the validity of the 3.1.4.11 JGI annotation.

An alternate explanation for SAFA missing an EC number annotation is that the PDB70 structural library is lacking structures representing the EC number. This is seen for Sphmag02G063800, which is annotated as 2.4.1.224 and 2.4.1.225 in the JGI file. There are no structures in the PDB70 structural library that are annotated with the 2.4.1.224 and 2.4.1.225 numbers. The SAFA primary EC number annotation is 2.4.1.223, derived from only two structural alignment hits. In this case, the disagreement between annotation methods is most likely to originate from the incompleteness of the structural library, although the SAFA annotation is still a partial success. If the structural library included structure representation of the missing EC numbers, its possible that the SAFA-derived EC number annotation would be 2.4.1.∗. All three relevant EC numbers for Sphmag02G063800 are related to the glycosaminoglycan biosynthesis - heparan sulfate / heparin pathway, indicating that SAFA is able to recover some relevant functional information even when the structural library is incomplete.

4 Comparing structural alignment against sequence-based alignment

Predicted structure-based alignments can provide more sensitive detection of structural motifs than can be achieved from sequence-based methods alone. Figure S8(A) depicts one example of this: the structural model of the Sphmag08G106500 primary transcript has average pLDDT and pTM scores of 73 and 0.63, respectively, representing a reasonably well-modeled protein. The JGI annotation information contains no evidence record for this primary transcript. The top hit from the HHblits alignments against the PDB70 sequence database has an E-value of 2.0, far and away from any significant homology. Furthermore, neither a BlastP nor HMMER alignment against non-redundant protein sequence databases resulted in matches to annotated proteins. Both methods do return some matches to proteins labeled as "uncharacterized" or "hypothetical" from other species. An HH-blits search against the UniRef30 also returns very few high-confidence alignments that are annotated, with no consensus among these few results. This protein is an example of the hardest proteins to annotate with purely sequence-based methods, even when using some of the most sensitive hidden Markov model-based approaches.

Using US-align2, six structural alignment hits were found in the PDB70 structural library, the best of which is depicted in panel (B) of Figure S8. The Sphmag08G106500 protein model is depicted in purple and chain A of PDB entry 2AV5 is depicted in green. The alignment has an average TM-score of 0.78 and an aligned sequence identity of 13.2%. The 2AV5 protein originates from *P. furiosus*, an archaeon, and the remaining structural alignment hits originate from other archaea as well. All six of the alignment hits are labeled as components of a ribonuclease P complex. No residue-level features are reported in the hits' UniProt flat files, as expected for a

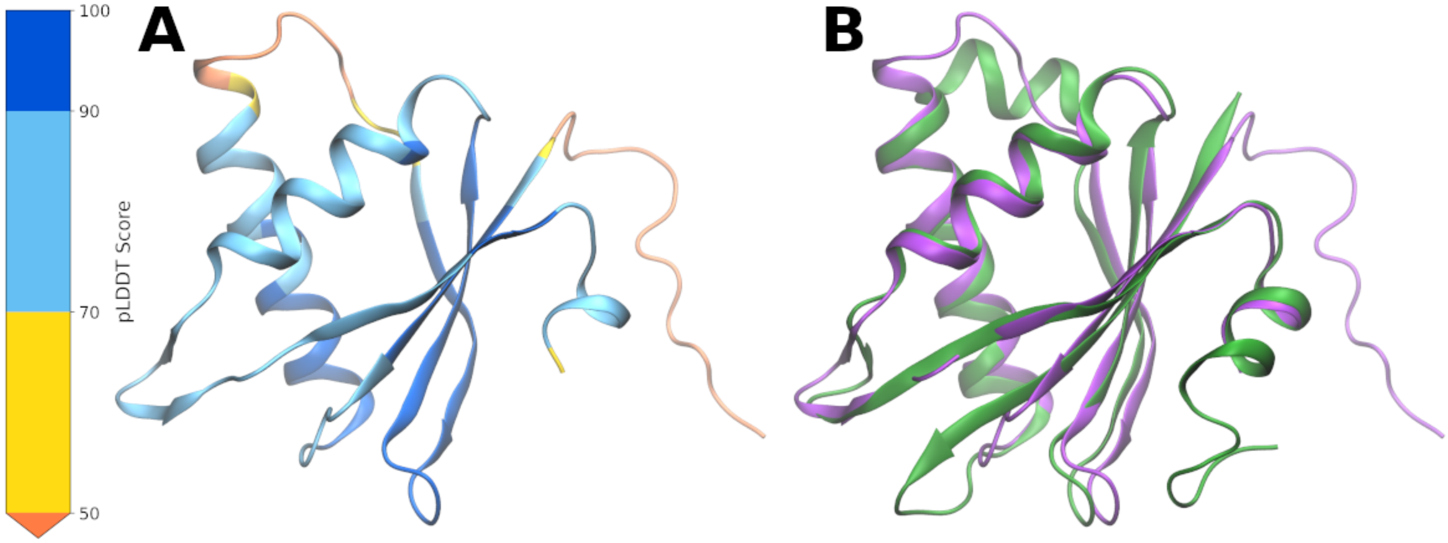

Figure S8: Structural model and top alignment for Sphmag08G106500, which may contain a ribonuclease P protein component structural motif. (A) pLDDT values visualized on the structure model. (B) Top structural alignment between the model (purple) and PDB entry 2AV5 (chain A).

protein that functions to structure and stabilize an RNA oligomer that performs the catalysis.

The structural motif of ribonuclease P proteins is detected with sequence-based methods in distant relatives of the *Sphagnum* genus, such as (*C. purpureus*), but the sequence differences in *S. divinum*'s Sphmag08G106500 seem to cross the threshold of detection for these approaches. Figure S9 shows the sequence alignment and sparse phylogenetic tree between this protein and homologs in *S. fallax* (angustifolium) and *C. purpureus*, as well as a paralog in *S. divinum*, Sphmag08G004200, illustrating the differences in the sequence that push it beyond detection. With the combination of AlphaFold prediction and US-align2 alignment against PDB70 structural library, we can find strong structural analogs for investigation and from which functional annotation hypotheses can be proposed.

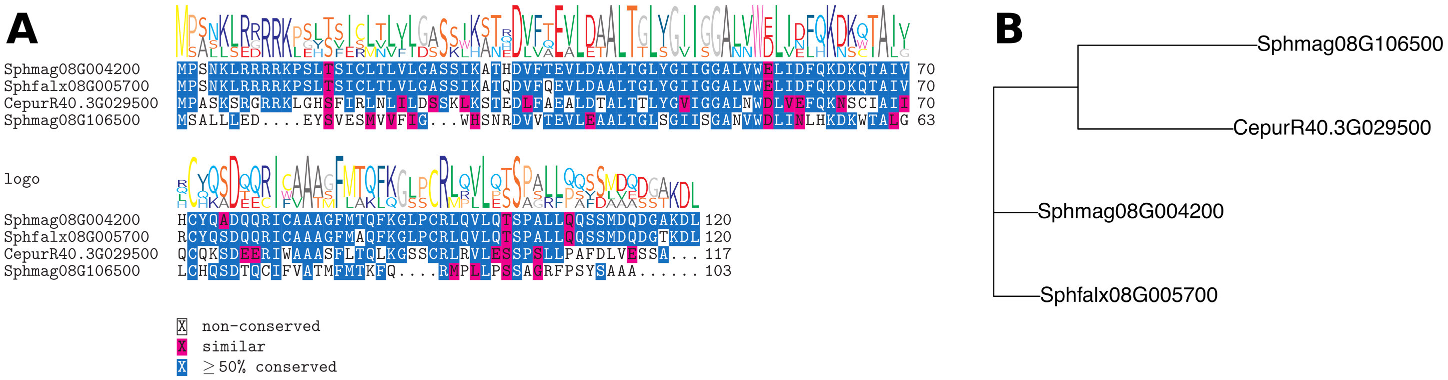

Figure S9: Alignment of protein sequences (A) and associated phylogenetic tree (B) of Sphmag08G106500, a paralog in *S. divinum* (Sphmag08G004200), and orthologs in S. fallax (angustifolium; Sphfalx08G005700) and *C. purpureus* (CepurR40.3G029500). For the sequence logo, conserved and similar amino acid residues are highlighted with dark blue and purple, respectively. The phylogenetic tree was generated using Neighbor-Joining method on the basis of the protein sequence alignment. The paralogs in *S. divinum* have a sequence identity of 56.% and have similar structural models and alignment hits. Discussed in more depth in Section S5.

4.1 Large-scale comparison

Whether in tandem or as an alternative to structural prediction and alignment, one might use a sequence-based alignment against a library of sequences that originate from a structural database (e.g., the PDB70); such a method may provide structural information that can be used to elucidate functional annotations.

To compare structural alignment of predicted structures against the state-of the-art remote homology detection with sequence-based alignments, we have used the HHblits program [5, 27] to align *S. divinum* protein sequences

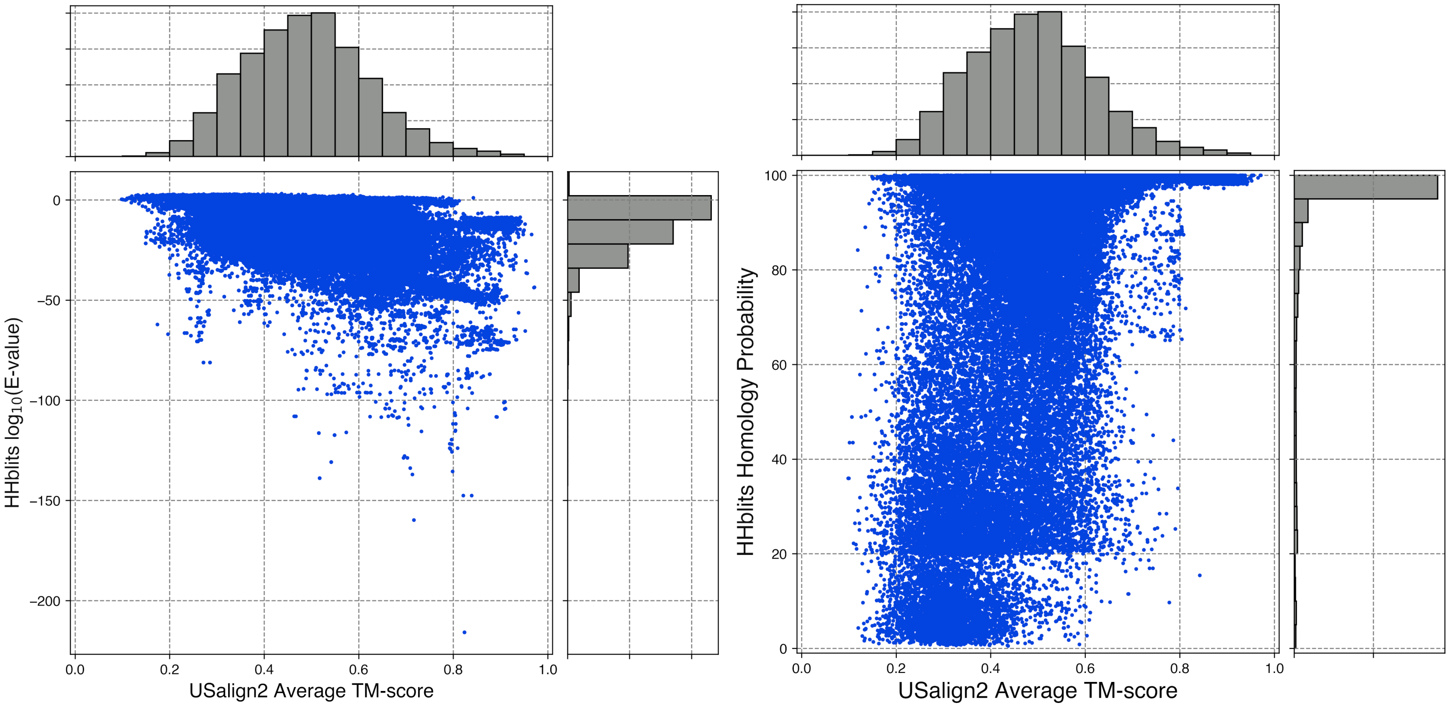

Figure S10: Scatter plots of the sequence and structural alignment results associated with the 1,104 heat-stress related proteins. Each of the 132,413 data points represents the alignment quality values from both methods associated with one pair of *S. divinum* protein and PDB70 entry, where the HHblits metric is $\log_{10}$(E-value) (left) and the homology probability (right).

against the PDB70 sequence library. For this comparison, we use the set of 1,104 structurally well-modeled proteins that have been highlighted as important to stress response through gene expression experiments. Many of these are also difficult to annotate confidently. Figure S10 shows the scatter plots and distributions of $\log_{10}$(E-value) and homology probability values from HHblits alignment versus US-align2's average TM-score results, where each scatter point represents the score of one model's alignment to one target in the associated PDB70 library. There does not appear to be a strong correlation between alignment scores produced from the two methods when considering the 132,413 data points of directly comparable model-target pairs.

In our structural alignment pipeline, we use a strict average TM-score cutoff of 0.7, which corresponds to both aligned structures having very similar three-dimensional placement of the protein backbone and thereby returning a very close structural match (e.g., the alignment depicted in Figure S8B). For sequence alignments using HHblits, alignment quality cutoffs of over 99% homology probability or an E-value less than $1e-10$ generally signify good scores for distant homology detection. We consider strong alignment results from both methods as defined by the sequence alignment E-value $< 1e-10$ and the associated structural alignment's average TM-score as > 0.70. 234 *S. divinum* proteins have at least one alignment target whose alignment quality metrics satisfy both cutoffs, which is 21% of the 1,104 stress-response proteins used as the comparison population. Overall, we see that for this dataset, a sensitive sequence-based alignment against the PDB70 sequence database may frequently fail to identify strong structural matches that are identified by structural alignment analysis.

An important caveat for this comparison is that data were only considered for primary transcripts that have high-quality structural models from AlphaFold. For proteins with low-confidence structural models, no US-align2 analysis was performed since the validity of any structural alignment results, whether good or bad, would be questionable at best. Errors propagating into a proteome's worth of functional annotation hypotheses should be minimized since experimental validation of one annotation requires extensive amount of work. For instances where a protein has a low quality structural model, whether inferred from AlphaFold 2 or other protein structure prediction methods, then the sequence alignment methods would be the next viable option for protein functional annotation using computational methods.

5 Sequence alignment and phylogenetic tree of putative ribonuclease P proteins

Amino acid sequences of Sphmag08G106500, its paralog (Sphmag08G004200), and orthologs in *S. fallax* (angustifolium) and *C. purpureus* were identified and obtained from Phytozome (https://phytozome-next.jgi.doe.gov/). Multiple sequence alignment and visualization was conducted using `msa` and `msaPrettyPrint` functions from msa v1.30.1 R package. The phylogenetic tree was produced by plot.phylo from ape v5.7.1 package by plotting Neighbor-joining tree generated from identity distance by `dist.alignment` from R seqinr v4.2.30 package.

6 Sphmag13G047200 - Hypothesized Cytochrome P450.

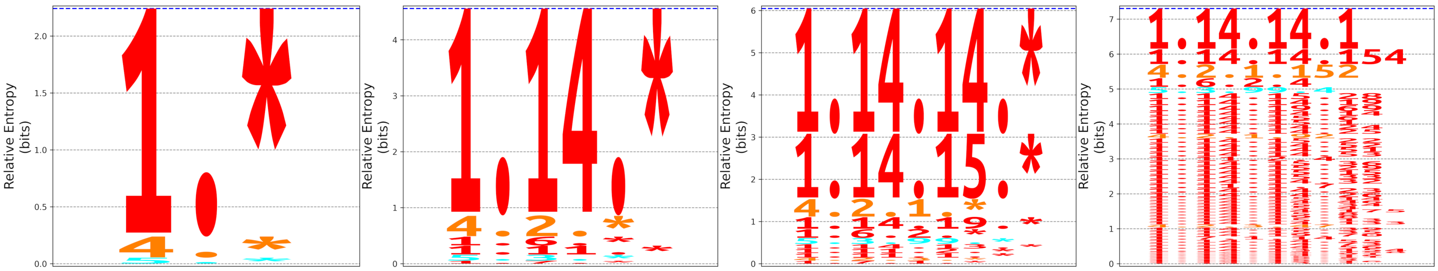

Figure S11: The series of relative entropy logo figures for (A) first digit, (B) second digit, (C) third digit EC prefixes as well as (D) full EC numbers. Sphmag13G047200 has 169 full EC numbers in its associated alignment hits' metadata, where a broad range of full EC numbers are observed. Considering the prefixes, the observed full EC numbers originate from only a few prefixes, where 1.∗, 1.14.∗, 1.14.14.∗ dominate the stacks in the respective panel. Consensus in the relative entropy components is obtained at the third digit prefix.

As discussed in the manuscript, the Sphmag13G047200 model aligns well to 309 structures in the PDB70 structural library. 300 of these targets have an associated UniprotKB ID, from which the metadata flat files are gathered and parsed. There are 228 flat files with an EC number in the description (DE) line; 59 of these have an incomplete or out-dated EC number that are not considered for the annotation analysis. For example, chain C of 3TDA is a cytochrome P450 from *H. sapiens* that is labeled with the 1.14.14.- EC number. This structure aligns strongly with the Sphmag13G047200 model, resulting in an average TMscore of 0.85. Although a strong structural alignment, the incomplete EC number is not considered for the annotation analysis due to considerations about quality control of incomplete or outdated EC numbers affecting the proposed annotations (see above for more discussion on this). Any incomplete EC numbers gathered from alignment hits may add support for a more complete enzymatic annotation developed from the active, full EC numbers.

Figure S11 shows the series of EC number logos obtained from the 169 active EC numbers. As seen in panel D, there is a large set of EC numbers observed in the ensemble of alignment hits, none with sufficient relative entropy components to form a consensus. Of note, there are four counts of the 5.3.994 EC number (prostaglandin-I synthase), 9 counts of 4.2.1.152 (hydroperoxy icosatetraenoate dehydratase), and 3 counts of 4.2.1.92 (hydroperoxide dehydratase); these are the only non-oxidoreductase categorized EC numbers observed. Even when such a broad set of EC numbers are observed, all directly correspond to cytochrome P450 or other heme-binding enzymes. In this way, all EC numbers associated with a model's alignment hits, whether incomplete or active, provide support for the hypothesis that the Sphmag13G047200 structure binds a heme cofactor, as shown in Figure 3 in the manuscript.

7 Dask-enabled workflow.

To automate the ∼1.3 billion alignment calculations between *S. divinum* structural models and the PDB70 structural library, a `dask.distributed` workflow was implemented on the OLCF Andes machine, using the default partition where each compute node has two 16-core 3.0 GHz AMD EPYC processors. The workflow was

tested on a wide range of compute allocations, ranging from one compute node, where the dask scheduler and client script were given one CPU core each with 30 workers, to 64 compute nodes, where the dask scheduler and client script run on one node and hundreds to thousands of workers are allocated the remaining resources. Wall clock time is very limited on OLCF Andes for batch jobs that request more than 64 compute nodes; this was the max allocation requested for the SAFA workflow and enabled the use of 2016 workers (63 nodes with 32 workers each). The same workflow has also been tested on a smaller scale using a desktop machine with an Intel Core i7-7700K Processor, where eight workers utilized one thread each. For both implementations, no optimizations of the US-align2 source code was implemented; the semi-nonsequential alignment algorithm was used for all alignments.

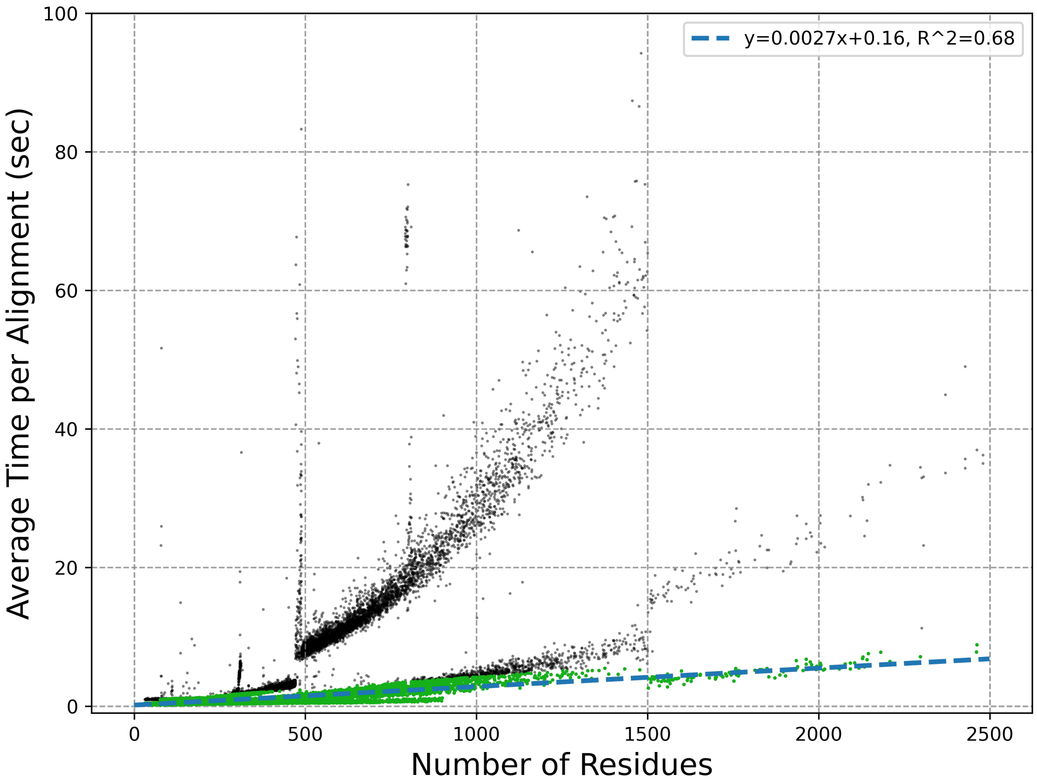

Figure S12: Timescaling data for the large scale SAFA workflow implementation on OLCF Andes. Instances of low performance for the *S. divinum* primary transcripts are shown in black while the "unaffected" data points are green. Linear regression of the unaffected data returns an estimator for total compute time for the 1.3 billion calculations, correlating the average time per alignment with the number of amino acid residues in the structural model. This is a rough approximation used to calculate computational efficiency as well as an estimate of total compute time.

7.1 SAFA workflow on HPC

Due to machine-wide IO issues on OLCF Andes during the time period of the large scale calculations, we do not have exact values for total compute time used nor accurate computational efficiency. A small, unaffected sample of the timescaling information of Andes calculations have been used to estimate an approximate time per alignment. A rudimentary linear regression of the average time for one alignment versus a structural model's number of residues is shown in Figure S12, where the "unaffected" data is shown in green while the remaining points in black are observed timing data during IO slow downs on the Andes file system. An iterative fitting of data was used to remove large outliers in timing data. Specifically, for each iteration, each data point's residual from the linear fit was calculated. If residuals were greater than the 95 percentile of the residual distribution, the associated data point was ignored for the next iteration's fit. Iteration was stopped once a fit's R^2 value decreased relative to the previous iteration's, after which the previous fit was used. Four iterations were performed, resulting

in the linear fit shown in Figure S12.

Using this regression, we can approximate the total compute time for each *S. divinum* high-quality structural model. For example, Sphmag13G047200 is 536 residues and is estimated to take a total of 40.1 hours in compute time to align with all 91,032 structures in the PDB70 structural library; once these tasks are distributed across 2,016 workers (63 compute nodes worth of workers), this takes 1.2 minutes. When scaled from a single protein to all 14,410 *S. divinum* primary transcripts with high-quality structural models, 19,736 compute days are required to perform the 1.3 billion calculations; when distributed across 2016 workers, this equates to 9.8 days of real time. We wish to emphasize that these numbers are estimates of efficiency observed from a small subset of proteins' calculations. Additionally, the average time per alignment metric accounts for the full dask workflow (start up, developing the dask task graph, running US-align2 alignments, post-processing of alignment results, and IO of results). When a large population of workers is used, overhead times increase due to software and hardware limitations: the Dask scheduler has limited communication channels and communications between nodes is limited by the interconnects and overall burden on the file system.

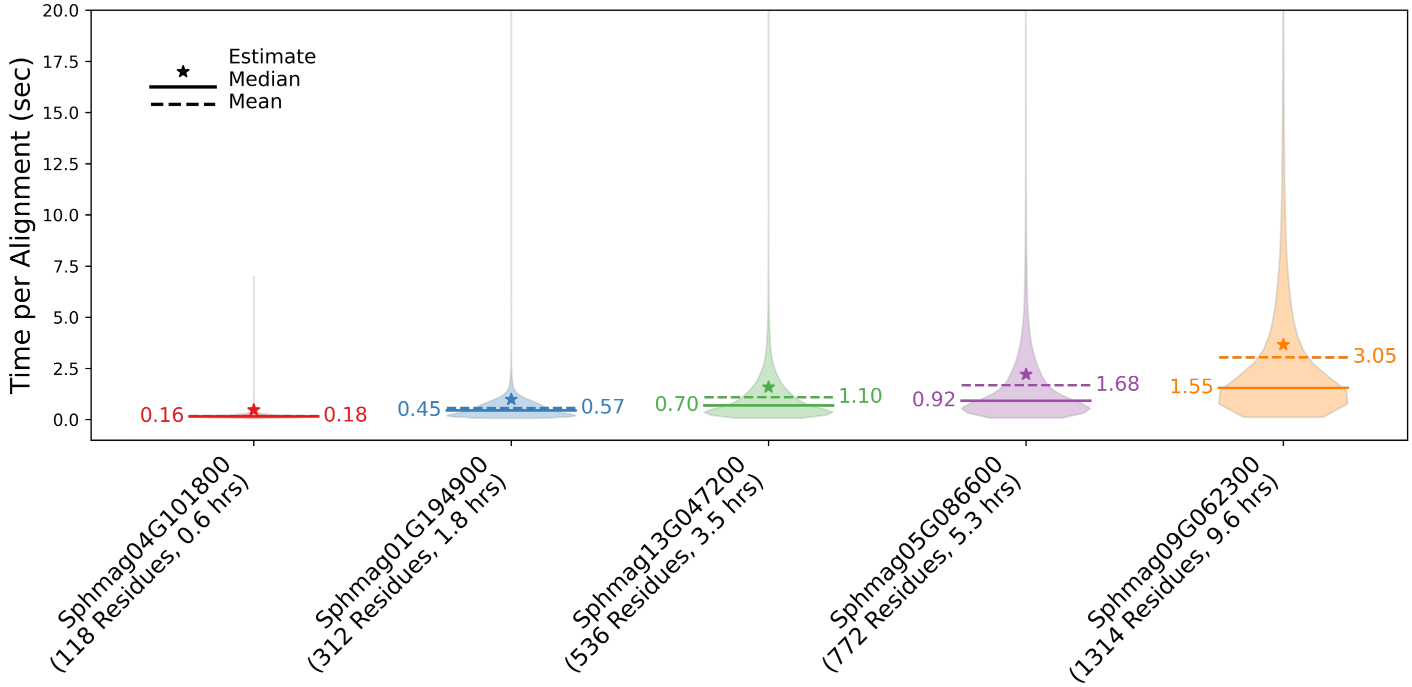

Figure S13: SAFA workflow timescaling data for five *S. divinum* structural models aligned to the PDB70 structural library, performed on a small desktop compute resource with an Intel Core i7-7700K Processor with 4 dual-threaded cores. Eight dask workers were utilized for all calculations shown here. For these runs, time per calculation for each alignment between the structural model and the PDB70 structural library is known. The median and mean values for the time per alignment are shown with solid and dashed line, respectively. Additionally, the model's number of residues is used to estimate the average time per alignment using the HPC-resource linear regression, shown with a star.

7.2 SAFA workflow on desktop computing resources

Alternatively to HPC resources, the SAFA workflow can be implemented on local or small-scale compute resources, such as the desktop computer described above. Five *S. divinum* structural models, spanning a range of number of residues, were aligned against the PDB70 structural library to give a sense of compute efficiency at smaller scale applications. Figure S13 visualizes the distributions of each structural models' 91,032 alignment calculation times, where the average and median for this distribution are depicted with dashed and solid lines. The real time to completion for the five structural models is shown in the x-axis labels. Additionally, the number of residues for each model is used to estimate the expected average time per calculation using the regression shown in Figure S12. This estimate is shown with a star for each model and is biased to longer times than the observed average times; this comparison is somewhat unfair since the two compute resources have different

hardware. For example, on the desktop computer, all alignment calculations for Sphmag13G047200 (green distribution) were observed to take 27.8 hours of compute time compared to the 40.1 hours on OLCF Andes. In real time, the OLCF Andes run for Sphmag13G047200 was estimated to take 1.2 minutes with 2,016 workers while the desktop computer finished all tasks in 209 minutes (~3.5 hours) with 8 workers.

7.3 Discussion

Generally, the application of `dask.distributed` for the SAFA workflow enables the massive amount of calculations to be performed in very reasonable times, whether on HPC or desktop compute resources. We do advise that SAFA applied to proteome-scale annotation campaigns (hundreds to thousands of structural models) utilize HPC resource allocations. Small scale compute resources can be utilized for small scale campaigns. Both applications utilize the same Python scripts with no changes required. See `https://github.com/BSDExabio/SAFA` for the code.

In regards to the estimates provided above, it is important to note that the mean of time per alignment shown in Figure S13 becomes a worse descriptor for the distributions as the number of residues in the target structure increases. We hypothesize this is true for the HPC resource timescaling data as well, suggesting that the computational efficiency and total compute time estimates reported in Section S7.1 may be overestimates of the real timing data if the HPC resource had performed ideally.

An additional consideration for computational efficiency of the SAFA workflow in regards for annotating enzymes is the number of structures in the PDB70 structural library that lacked any enzymatic annotation information. Figure S6 shows that only 26,455 of the structures in the full library had explicitly been annotated with EC numbers, just under 30% of the library. To the loss of non-EC number metadata, the SAFA workflow could be applied to align only this subset of structures; this would drastically decrease the total compute time required. A list of enzyme structures in the PDB70 structural library is provided in the data repository at `https://doi.org/10.5281/zenodo.7953087`.

References

[1] W Humphrey, A Dalke, and K Schulten. "VMD: visual molecular dynamics." In: *J. Mol. Graph.* 14.1 (1996), pp. 33–8, 27–8.

[2] John Jumper, Richard Evans, Alexander Pritzel, et al. "Highly accurate protein structure prediction with AlphaFold". In: *Nature* 596.7873 (2021), pp. 583–589.

[3] Mu Gao, Mark Coletti, Russell B. Davidson, et al. "Proteome-scale deployment of protein structure prediction workflows on the Summit supercomputer". In: *2022 IEEE International Parallel and Distributed Processing Symposium Workshops (IPDPSW)*. IEEE, 2022, pp. 206–215. eprint: 2201.10024.

[4] Timo Lassmann and Erik LL Sonnhammer. "Kalign - An accurate and fast multiple sequence alignment algorithm". In: *BMC Bioinform.* 6.1 (2005), pp. 1–9. DOI: 10.1186/1471-2105-6-298.

[5] Martin Steinegger, Markus Meier, Milot Mirdita, et al. "HH-suite3 for fast remote homology detection and deep protein annotation". In: *BMC Bioinform.* 20.1 (2019), pp. 1–15.

[6] Lorna Richardson, Ben Allen, Germana Baldi, Martin Beracochea, Maxwell L. Bileschi, Tony Burdett, Josephine Burgin, Juan Caballero-Perez, Guy Cochrane, Lucy J. Colwell, Tom Curtis, Alejandra Escobar-Zepeda, Tatiana A. Gurbich, Varsha Kale, Anton Korobeynikov, Shriya Raj, Alexander B. Rogers, Ekaterina Sakharova, Santiago Sanchez, Darren J. Wilkinson, and Robert D. Finn. "MGnify: the microbiome sequence data analysis resource in 2023". In: *Nuc. Acids Res.* 51.D1 (2023), pp. D753–D759. DOI: 10.1093/nar/gkac1080.

[7] Milot Mirdita, Lars Von Den Driesch, Clovis Galiez, Maria J. Martin, Johannes Soding, and Martin Steinegger. “Uniclust databases of clustered and deeply annotated protein sequences and alignments”. In: *Nuc. Acids Res.* 45.D1 (2017), pp. D170–D176. DOI: 10.1093/nar/gkw1081.

[8] Peter Eastman, Jason Swails, John D. Chodera, Robert T. McGibbon, Yutong Zhao, Kyle A. Beauchamp, Lee Ping Wang, Andrew C. Simmonett, Matthew P. Harrigan, Chaya D. Stern, Rafal P. Wiewiora, Bernard R. Brooks, and Vijay S. Pande. “OpenMM 7: Rapid development of high performance algorithms for molecular dynamics”. In: *PLoS Comput. Biol.* 13 (2017), e1005659.

[9] Viktor Hornak, Robert Abel, Asim Okur, Bentley Strockbine, Adrian Roitberg, and Carlos Simmerling. “Comparison of multiple Amber force fields and development of improved protein backbone parameters”. In: *Proteins* 65.3 (2006), pp. 712–725.

[10] Richard J. Gowers, Max Linke, Jonathan Barnoud, Tyler J. E. Reddy, Manuel N. Melo, Sean L. Seyler, Jan Domański, David L. Dotson, Sébastien Buchoux, Ian M. Kenney, and Oliver Beckstein. “MDAnalysis: A Python package for the rapid analysis of molecular dynamics simulations”. In: *Proceedings of the 15th Python in Science Conference*. SciPy, 2016, pp. 98–105.

[11] Naveen Michaud-Agrawal, Elizabeth J. Denning, Thomas B. Woolf, and Oliver Beckstein. “MDAnalysis: A toolkit for the analysis of molecular dynamics simulations”. In: *J. Comput. Chem.* 32.10 (2011), pp. 2319–2327.

[12] The UniProt Consortium. “UniProt: the Universal Protein Knowledgebase in 2023”. In: *Nucleic Acids Res.* 51.D1 (2023), pp. D523–D531.

[13] Stephen F. Altschul, Warren Gish, Webb Miller, Eugene W. Myers, and David J. Lipman. “Basic local alignment search tool”. In: *J. Mol. Biol.* 215.3 (1990), pp. 403–410.

[14] Sameer Velankar, José M. Dana, Julius Jacobsen, Glen Van Ginkel, Paul J. Gane, Jie Luo, Thomas J. Oldfield, Claire O’Donovan, Maria Jesus Martin, and Gerard J. Kleywegt. “SIFTS: Structure Integration with Function, Taxonomy and Sequences resource”. In: *Nuc. Acids Res.* 41.D1 (2013), pp. D483–D489. DOI: 10.1093/nar/gks1258.

[15] Jose M. Dana, Aleksandras Gutmanas, Nidhi Tyagi, Guoying Qi, Claire O’Donovan, Maria Martin, and Sameer Velankar. “SIFTS: Updated Structure Integration with Function, Taxonomy and Sequences resource allows 40-fold increase in coverage of structure-based annotations for proteins”. In: *Nuc. Acids Res.* 47.D1 (2019), pp. D482–D489. DOI: 10.1093/nar/gky1114.

[16] I. Schomburg, L. Jeske, M. Ulbrich, S. Placzek, A. Chang, and D. Schomburg. “The BRENDA enzyme information system–From a database to an expert system”. In: *J. Biotechnol.* 261 (2017). Bioinformatics Solutions for Big Data Analysis in Life Sciences presented by the German Network for Bioinformatics Infrastructure, pp. 194–206.

[17] Antje Chang, Lisa Jeske, Sandra Ulbrich, Julia Hofmann, Julia Koblitz, Ida Schomburg, Meina Neumann-Schaal, Dieter Jahn, and Dietmar Schomburg. “BRENDA, the ELIXIR core data resource in 2021: new developments and updates”. In: *Nucleic Acids Res.* 49.D1 (2020), pp. D498–D508.

[18] Chengxin Zhang, Morgan Shine, Anna Marie Pyle, and Yang Zhang. “US-align: universal structure alignments of proteins, nucleic acids, and macromolecular complexes”. In: *Nat. Methods* 19.9 (2022), pp. 1109–1115.

[19] Chengxin Zhang and Anna Marie Pyle. “A unified approach to sequential and non-sequential structure alignment of proteins, RNAs and DNAs”. In: *iScience* 25.10 (2022), p. 105218.

[20] Alexej Abyzov and Valentin A. Ilyin. “A comprehensive analysis of non-sequential alignments between all protein structures”. In: *BMC Structural Biology* 7.1 (2007), pp. 1–20.

[21] Matthew Rocklin. “Dask: Parallel computation with blocked algorithms and task scheduling”. In: *Proceedings of the 14th Python in Science Conference (SciPy 2015)*. Vol. 130. SciPy Austin, TX. 2015, p. 136.

[22] Yang Zhang and Jeffrey Skolnick. "Scoring function for automated assessment of protein structure template quality". In: *Proteins* 57.4 (2004), pp. 702–710.

[23] Thomas D. Schneider and R. Michael Stephens. "Sequence logos: A new way to display consensus sequences". In: *Nucleic Acids Res.* 18.20 (1990), pp. 6097–6100.

[24] Gavin E. Crooks, Gary Hon, John Marc Chandonia, and Steven E. Brenner. "WebLogo: A sequence logo generator". In: *Genome Res.* 14.6 (2004), pp. 1188–1190.

[25] J. D. Hunter. "Matplotlib: A 2D graphics environment". In: *Comput. Sci. Eng.* 9.3 (2007), pp. 90–95. DOI: 10.1109/MCSE.2007.55.

[26] Alex Clark. *Pillow (PIL fork) documentation*. 2015.

[27] Michael Remmert, Andreas Biegert, Andreas Hauser, and Johannes Söding. "HHblits: Lightning-fast iterative protein sequence searching by HMM-HMM alignment". In: *Nat. Methods* 9.2 (2012), pp. 173–175.

[28] Adam L Healey, Bryan Piatkowski, John T Lovell, et al. "Newly identified sex chromosomes in the Sphagnum (peat moss) genome alter carbon sequestration and ecosystem dynamics". In: *Nat. Plants* (2023), pp. 1–17.

[29] P. M. Hayward, R. S. Clymo, and Gordon Elliott Fogg. "Profiles of water content and pore size in <i>Sphagnum</i> and peat, and their relation to peat bog ecology". In: *Proceedings of the Royal Society of London. Series B. Biological Sciences* 215.1200 (1982), pp. 299–325.

[30] Rob Patro, Geet Duggal, Michael I Love, Rafael A Irizarry, and Carl Kingsford. "Salmon provides fast and bias-aware quantification of transcript expression". In: *Nature methods* 14.4 (2017), pp. 417–419.

[31] Michael I Love, Wolfgang Huber, and Simon Anders. "Moderated estimation of fold change and dispersion for RNA-seq data with DESeq2". In: *Genome biology* 15.12 (2014), pp. 1–21.
